# RUNX transcription factors are essential in maintaining epididymal epithelial differentiation

**DOI:** 10.1007/s00018-024-05211-5

**Published:** 2024-04-17

**Authors:** Mervi Toriseva, Ida Björkgren, Arttu Junnila, Arfa Mehmood, Jesse Mattsson, Inka Raimoranta, Bongki Kim, Asta Laiho, Matthias Nees, Laura Elo, Matti Poutanen, Sylvie Breton, Petra Sipilä

**Affiliations:** 1 Institute of Biomedicine, Cancer Research Unit and FICAN West Cancer Centre Laboratory, University of Turku and Turku University Hospital, Turku, Finland; 2 Institute of Biomedicine, Research Centre for Integrative Physiology and Pharmacology, Turku Center for Disease Modeling, University of Turku, Turku, Finland; 3 Turku Bioscience Centre, University of Turku and Åbo Akademi University, Turku, Finland; 4 Program in Membrane Biology/Division of Nephrology, Massachusetts General Hospital, Simches Research Center, Boston, MA 02114 USA; 5 Institute of Medicine, The Sahlgrenska Academy, Gothenburg University, Göteborg, Sweden; 6 Department of Animal Resources Science, Kongju National University, Chungcheongnam-do, Yesan, 32439 Republic of Korea; 7 Department of Obstetrics, Gynecology and Reproduction, Faculty of Medicine, Research Center-CHU de Québec, Université Laval, Québec, QC Canada

**Keywords:** Epididymis, RUNX1, RUNX2, Epithelial to mesenchymal transition, MAPK signaling, NOTCH, Loss of epithelial phenotype, Development

## Abstract

Apart from the androgen receptor, transcription factors (TFs) that are required for the development and formation of the different segments of the epididymis have remained unknown. We identified TF families expressed in the developing epididymides, of which many showed segment specificity. From these TFs, down-regulation of runt related transcription factors (RUNXs) 1 and 2 expression coincides with epithelial regression in Dicer1 cKO mice. Concomitant deletion of both *Runx1* and *Runx2* in a mouse epididymal epithelial cell line affected cell morphology, adhesion and mobility in vitro. Furthermore, lack of functional RUNXs severely disturbed the formation of 3D epididymal organoid-like structures. Transcriptomic analysis of the epididymal cell organoid-like structures indicated that RUNX1 and RUNX2 are involved in the regulation of MAPK signaling, NOTCH pathway activity, and EMT-related gene expression. This suggests that RUNXs are master regulators of several essential signaling pathways, and necessary for the maintenance of proper differentiation of the epididymal epithelium.

**Supplementary Information:**

The online version contains supplementary material available at 10.1007/s00018-024-05211-5.

## Introduction

The mammalian epididymis is necessary for post-testicular sperm maturation [1, 2] and several genetically modified mouse models have shown that a lack or dysfunction of the proximal epididymal epithelium leads to male infertility [3]. Thus, understanding epididymal development, especially the segmentation of the epididymal duct and driver transcription factors (TFs) involved, is of great importance. During embryogenesis, the epididymis develops from the mesonephric tubules and the proximal Wolffian duct (WD) [4, 5]. After birth, the undifferentiated epididymal epithelium first evolves through a proliferative phase. In rodents this continues until around P15, and the following differentiation of various epithelial cell types and segment identities is completed around P44 [6, 7]. The fully differentiated mouse epididymis is composed of four segments: initial segment (IS), caput, corpus and cauda, which form a pseudostratified epithelial layer composed of different cell types: principal, narrow/clear and basal cells. Each of the epididymal segments has a unique gene expression pattern and functions to ensure proper sperm maturation [1–3].

TFs are master regulators in tissue differentiation and the maintenance of cellular identity. Previous work identifying mouse TFs from 24 adult tissues and 8 fetal tissues revealed that in each tissue, the four most abundant TFs account for over 30% of the total TF amount in that given tissue [8]. Furthermore, from approximately 1600 TFs encoded by the mammalian genome, only a few dozen are necessary for the full development of many tissues [9–11] and a strikingly small number of TFs are required for directing pluripotent stem cells toward various differentiated cell types such as neurons, hepatocytes and pancreatic beta-cells [12]. In the epididymis, androgen receptor (AR) signaling has a prominent and well-known role in the development of the organ. During embryonal development, AR expression in the mesenchyme surrounding the WD is required for stabilization, elongation, and coiling of the WD, whereas AR signaling in the WD epithelium is required for proper differentiation of epididymal principal and basal cells [13]. Conditional deletions (cKO) of the AR in the proximal epithelium later during development have demonstrated that epithelial AR signaling is a prerequisite for the formation of IS and differentiation of various epithelial cell types [14, 15]. Whereas AR function is critical for principal and basal cell differentiation, apical and clear cells of the proximal epididymis seem to depend on estrogen receptor 1 (ESR1) function [16]. However, apart from the AR, the TFs that are required for the development of the epididymis and the formation of the segment identity of epididymal segments, are not known.

We have previously generated a mouse model, where *Dicer1* was conditionally deleted from the proximal epididymis, initial segment and caput, at P12 [17]. The lack of DICER1 caused the loss of proper epithelial cell identity of the epididymal epithelium around the age of 5 weeks, imbalance in sex steroid signalling [17], and subsequent male infertility due to undifferentiated IS and caput region [18]. To our best knowledge, this model is the only one in which the differentiation of the epithelium first begins, but is subsequently halted, and finally the epithelium is regressed into a more undifferentiated state. As DICER1 is an essential RNAse III enzyme in the miRNA processing pathway [19], it likely affects a wide variety of signaling pathways in the epididymis.

In this study, we aimed to identify the TFs that are necessary for maintenance of the differentiated epididymal epithelium in the mouse proximal epididymis, and thus examined the gene expression profile in Dicer1 cKO epididymides at three different ages during development with RNA-seq. We identified a number of TF families that were presented in the developing epididymides, of which many showed segment-specific expression. From these TFs, we identified one family in which the down-regulation of expression coincides with observed histological changes in Dicer1 cKO epididymal epithelium, namely runt related transcription factors (RUNXs) 1 and 2. We then showed that deletion of either *Runx1* or *Runx2,* or concomitant deletion of both in the mouse epididymal cell line mE-Cap18 [20], affected cell adhesion in vitro. However, only the concurrent deletion of *Runx1* and *Runx2* severely defected the formation of epididymal organoid-like structures in 3D cell cultures. Transcriptomic analysis of these structures suggested that RUNX1 and RUNX2 are involved in the control of several essential signaling pathways and epithelial cell plasticity, and thus are necessary for the maintenance of proper differentiation of the epididymal epithelium.

## Materials and methods

### Mouse model

Conditional knockout (cKO) mice of *Dicer1* in the proximal part of the mouse epididymis (Dicer 1 cKO mice) used in the study, have been described earlier [17, 18]. Littermate homozygous Dicer1 flox were used as control animals (Ctr). The mice were housed in individually ventilated cages under controlled conditions of light, temperature, and humidity at the specific pathogen fee (SPF) unit of Central Animal Laboratory of University of Turku, Finland. All animals received a soy-free SDS-RM3 diet (Special Diets Service, Witham Essex, United Kingdom), and tap water and chow were available ad libitum. Animal experiments were conducted with the approval of the Finnish Animal Ethics Committee and also fully met the requirements as defined by the U.S. National Institutes of Health guidelines on animal experimentation. For in vitro experiments, male mice were killed at different age points, 25, 35 or 40 days of age, using carbon dioxide asphyxiation and cervical dislocation. Collected tissues were weighed and snap frozen in liquid nitrogen or fixed in formalin for histological analyzes. In addition, WT C57Bl/6NRj males (Janvier Labs) were used to analyze *Runx1* and *Runx2* expression during the development and different epididymal segments from adult males. Epididymides were collected at the ages of 14, 21, 28 and 42 days as well as 2 months. From adult epididymides IS, caput, corpus and cauda epididymidis were separated before snap frozen in liquid nitrogen.

### Cell lines

An immortalized mouse proximal epididymal (mE-Cap18) cell line [20] was utilized to study the role of Runx transcription factors for epididymal epithelial cell functions. All CRISPR-Cas9 reagents were obtained from Integrated DNA technologies (IDT). Prior to transfection, mE-Cap18 cells were seeded at 80,000 cells/well into 6-well plates without antibiotics. Cells were then transfected with Cas9:crRNA:tracrRNA ribonucleoprotein complex: 60 nmol Alt-R™ S.p. Cas9 endonuclease, 60 nmol Alt-R™ CRISPR tracrRNA, and 30 nmol of each of the two target specific Alt-R™ CRISPR crRNAs (for deleting exon 5 from Runx1: Runx1e5 3A GAAGTAAGTGAGCCCCCTTG and Runx1e5 5A CAGAGTGAAGCTCTTGCCTG; for deleting Runx2 exon 4 Runx2e4 GTAGGTTGTAGCCCTCGGAG and Runx2e4 3A TTTGTGGGCCGGAGCGGACG) or Alt-R™ CRISPR Negative Control crRNA #1, prepared according to manufacturer’s instructions using 38 µl Lipofectamine^®^ RNAiMax (Thermo Fisher). Cells were incubated for 48 h at 37 °C in 5% CO_2_ before being sorted using BD FACSAria II cell sorter to obtain single-cell sorted clones. Cell clones were screened for deletion of Runx1 exon 5 and/or Runx2 exon 4 deletion by PCR using primers: Runx1 Fw1 ctggacagcatagactgacat + Runx1 Re1 gccacagatacattgtgagacc and Runx2 Fw1 agacaccatttacagggagca + Runx2 Re1 gcttgcaccagagagcctaa. PCR products smaller than from WT cells were sequenced to ensure deletion. In the case of *Runx1* inactivation, from 85 screened cell clones, one had complete exon 5 deletion in both alleles (Fig. S1) named mE-Cap18 dR1. For *Runx2*, screening of 93 clones resulted in one clone with a 42 bp deletion within exon 4 in one allele and a deletion of 155 bases of the altogether 157 bases long exon 4 in the other allele (Fig. S1), called mE-Cap18 dR2. For simultaneously inactivating both *Runx1* and -*2*, from a total of 75 clones, only one had a complete deletion of exon 5 of *Runx1* in both alleles and a 138 bp deletion in exon 4 of *Runx2* in one allele. The other allele of *Runx2* had two bases missing, leading to the disruption of the open reading frame and a premature stop codon (Fig. S1). The lack of *Runx1* and/or *Runx2* mRNA and protein was further confirmed by RT-qPCR and immunoblotting from the cell clones.

mE-Cap18 cell lines with mutations in *Runx1*, *Runx2* or both *Runx1* and *Runx2* genes (dR1, dR2 or ddR1 + R2, respectively) and the control cell lines (WT and Ctr) were maintained in a humidified incubator with 5% CO_2_ and 37 °C and cultured in DMEM/F12 (Sigma Aldrich) supplemented with 10% heat-inactivated fetal bovine serum (iFBS, Gibco), 2 mM l-glutamine (Gibco), 1% penicillin-streptomycin (Gibco).

### Histological analysis

Epididymal histology was analyzed at 25, 35 and 40 days of age. Epididymides were collected from mice in each age group, fixed in formalin at room temperature for o/n, dehydrated and embedded in paraffin. Paraffin blocks were sectioned at 5 μm thickness and stained with hematoxylin and eosin.

### Immunofluorescent staining of tissue sections

Immunofluorescent staining of TJP1, TJP2, TJP3, CLDN1, CLDN3 and CLDN4 to the Dicer1 cKO epididymides was performed as described previously [21]. Shortly lysine-paraformaldehyde-fixed tissues were cryoprotected by incubating them in a solution of 30% sucrose in PBS for at least 24 h, and then embedded in OCT compound (Tissue-Tek; Sakura Finetek) and frozen. Cryosections were dehydrated in PBS and heated by microwaving in an alkaline buffer (Vector Laboratory) for antigen retrieval. To block unspecific antibody binding sections were treated by 1% bovine serum albumin in PBS for 30 min at RT. Subsequently, the sections were incubated with primary antibody solution with 1:10 dilution of TJP1 antibody or 1:200 dilution of other antibodies, overnight at + 4 °C in a moist chamber and with secondary antibody (60′ RT). After each incubation the sections were washed with PBS. DAPI nuclear dye was used as a counter stain. The primary and secondary antibodies were diluted in DAKO antibody diluent (DAKO) and are listed in Table S1.

For immunofluorescent staining of RUNX1, RUNX2 and vimentin (VIM), formalin-fixed paraffin-embedded tissue sections were dehydrated, treated for antigen retrieval with 10 mM citrate buffer (pH 6.0) and then with 10% BSA-PBS-Tween 0.1% (1 h, RT) to block unspecific antibody binding. Subsequently, the sections were incubated with primary antibody solution with 1:100 dilution (o/n, + 4 °C) for all the primary antibodies, and with secondary antibody (1 h RT). After each incubation the sections were washed with PBS. DAPI (Sigma-Aldrich) nuclear dye was used as a counter stain. Immunofluorescent stainings were scanned with Pannoramic MIDI slide scanner (3DHISTECH). The primary and secondary antibodies used in this study are listed in Table S1.

### Organotypic 3D cell cultures

The medium used in all organotypic 3D cultures was DMEM/F12 supplemented with 15% iFBS, 5% l-glutamine, 2.5% Pen-Strep, 1 μg/ml hydrocortisone, 0.2 U/ml insulin, 0.1 nmol/l cholera toxin and 25 ng/ml EGF (modified from [22]). In indicated experiments, the medium was further supplemented with Slit2 recombinant protein (0.5 µg/ml).

For imaging purposes and to perform cell viability assay, organotypic 3D cell culturing was performed primarily as described previously [23]. Briefly, cells were seeded as single cells between growth factor reduced Matrigel layers (Corning #356231) on 96-well angiogenesis µ-plates (Ibidi GmbH) in the density of 2000 cells/well. After Matrigel polymerization, medium was gently added on the top and replaced with fresh medium every 2–3 days. The formation of organoid-like structures was followed up to 12 days.

To extract RNA and protein lysates from organotypic 3D cell cultures, the cells were suspended in Matrigel to final concentration of 4 mg/ml and in the density of 250,000 cells/ml, and seeded on pre-heated tissue culture plates as drops (vol 80 µl). The dishes were first kept up-side down for 30 min in 37 °C until Matrigel was solidified. Subsequently, the medium was gently added to cover the drops and it was changed every 2–3 days during the 12 days culturing period.

### Cell viability assay in organotypic 3D cultures

Organotypic 3D culture was performed in 96-well angiogenesis µ-plate platform as described above. The number of cells over time in a well was measured by quantitative analysis of metabolic activity with WST-8 (Cell Counting Kit-8 Kit, Dojindo) after culturing the cells for 1, 5, 7, 10 and 12 days. At each timepoint, 6 sample wells on the culture plate were analyzed. Here, the medium was aspirated and freshly prepared and prewarmed media containing 10% v/v WST-8 was added. After two hours incubation at 37 °C, the absorbance was measured at 450 nm with a Victor2 1420 Multilabel counter (PerkinElmer Wallac).

### Adhesion and proliferation measurements

Cells were seeded on 96-well plate and the adhesion of cells was monitored using real-time imaging with IncuCyte S3 (Sartorius) (10× objective). With the help of Image J Cell counter tool, the cells were counted from four exported images in four replicate wells for each cell type, at timepoints 30 min, 1 h, 3 h, 5 h from seeding (appr. 500 cells/image). Cells were manually classified based on their morphology to either round or spread cells corresponding to non-attached or attached cells, respectively. The data was visualized using GraphPad Prism 9 software.

For proliferation measurements, Nuclight Rapid Red dye (Sartorius) was added to the cell suspension for live-cell nuclear labeling before seeding the cells to 96-well plate according to manufacturer’s instructions. Proliferation of cells was monitored using real-time imaging with IncuCyte S3 (10×  objective, 2 h imaging interval) and analyzed based on nuclear counts using IncuCyte S3 software version 2020A. The data was visualized using GraphPad Prism 9 software.

### Cell motility assay

To test the motile capacity of cells, they were subjected to Matrigel invasion assay. For this purpose, 96-well ImageLock plates were first coated with 100 μg/ml Matrigel (Corning) diluted in cell culture medium and incubated in 37 °C for o/n, after which the cells were seeded on top to high confluency. On the following day, scratch wounds were made in the cell layers using the WoundMaker tool (Sartorius). Subsequently, a 50 µl aliquot of 4 mg/ml Matrigel diluted in cell culture medium was applied to provide a 3D matrix on the cell cultures. Plates were incubated in 37 °C for 4 h and cell culture media containing 10% iFBS was gently added on top. Subsequent wound closure was monitored using real-time imaging (10× objective and wide field mode, 2 h imaging interval) and analyzed using IncuCyte S3 software. The data was visualized using GraphPad Prism 9 software.

### RNA isolation from tissue samples and gene expression profiling

For RNA-seq, total RNA was isolated from the whole epididymides of 25-day-old and 35-day-old and from IS and caput of 40-day-old Dicer1 cKO and Ctr males (n = 3 for each genotype in each time point) by using Trisure reagent (Bioline, USA) according to manufacturer’s instruction. The quality of RNA was determined by spectrophotometry and Bioanalyzer. RNA samples were processed at the Finnish Functional Genomics Centre at the Turku Bioscience (formerly Turku Centre for Biotechnology) using Illumina TruSeq Stranded mRNA Sample Preparation Kit and sequenced with HiSeq 2500 system (Illumina, USA) using 50 bp read length and single end sequencing chemistry. The quality of the sequencing reads was checked with FastQC tool (v. 0.10.1) and were aligned to UCSC mm9 reference genome downloaded from Illumina iGenomes site (https://support.illumina.com/sequencing/sequencing_software/igenome.html) using TopHat (v. 2.0.10) [24] with default settings. Reads were assigned to RefSeq genes and counted using HTSeq (v. 0.5.4p3) [25]. Read counts were normalized using Trimmed Mean of the M values (TMM) method implemented in the edgeR R/Bioconductor package (R version 3.2) [26–28]. The statistical testing between sample groups was carried out using Limma package with voom transformation [29] and differentially expressed (DE) genes were selected based at FDR < 0.001 (calculated using Benjamini-Hochberg method) and fold-change > 2. Pheatmap R package (R version 3.6.1) was used for producing the heatmaps, using Ward’s method with Euclidean distance. Pathway analyzes were done using Metascape [30]. In order to analyze TFs expressed in the mouse epididymis, we used a list of TFs from A mouse tissue transcription factor atlas [8]. Mouse TFs were further divided to the major TF families according their DNA binding domains using The Human Transcription Factors database (http://humantfs.ccbr.utoronto.ca/ [31]). The TFs whose RNA-seq values at least in three independent samples was higher than the median of the entire sample set, were accounted to be expressed in the mouse epididymis.

### RNA isolation and RNA-seq from organotypic 3D cultures

To isolate RNA from 3D cultures, the organoids were harvested on day 12 of culture from Matrigel droplets grown on 24-well plates by using ice cold 5 mM EDTA-PBS. Replicate droplets were collected, combined and incubated on ice in a large volume of buffer until the Matrigel had dissolved. The solution containing soluble Matrigel and the organoids was centrifuged (100×*g*, 5 min, 4 C) and the pellet was washed once with ice cold PBS. Total RNA was isolated from cell pellets by using Trisure reagent (Bioline, USA) according to manufacturer’s instruction. The quality of RNA was determined by spectrophotometry and Bioanalyzer. RNA samples were collected from five independent experiments for Ctr and ddR1 + R2 cells.

RNA-seq was performed by Novogene Co. After library preparation and sequencing with Illumina NovaSeq 6000, data analysis was performed using a combination of programs; Read alignment to mm10 reference genome, downloaded from genome website browser (NCBI), was performed using STAR (v2.5). HTSeq v0.6.1 was used to count the reads mapped to each gene. The statistical testing between sample groups was carried out using the DESeq2 R package (2_1.6.3). The resulting p values were adjusted using the Benjamini and Hochberg’s approach for controlling the False Discovery Rate (FDR). Genes with an adjusted p value < 0.05 found by DESeq2 were assigned as differentially expressed. Pathway analyzes were done using Metascape, and GSEA MSigDB database [32, 33] was used to analyze potential enrichment of different Hallmark gene sets using set of down-regulated DE genes (log2FC < − 1.5, p ≤ 0.05).

### RT-qPCR

The RNA samples from WT epididymides were treated with DNase using DNase Amplification Grade Kit (Sigma-Aldrich) and 0.5 µg of RNA was used for cDNA synthesis using SensiFAST cDNA synthesis kit (Bioline). The cDNA samples were then used for quantitative PCR (qPCR) reactions. All samples were run in duplicate reactions. The RT-qPCR analyzes were carried out for the *Runx1* and *Runx2* with the primers: Runx 1 Fw: GCCATCAAAATCACAGTG, Runx1 Rev: GCTGAGGGTTAAAGGCAG, Runx2 Fw: AGATGGGACTGTGGTTAC and Runx2 Re: GGACCGTCCACTGTCACT. The CFX96 real-time PCR detection system (Bio-Rad) and SYBR Green (Thermo Fischer Scientific) were used for analyzes. The results were normalized to ribosomal protein L19 (L19 Fw: GGACAGAGTCTTGATGATCTC and L19 Rev: CTGAAGGTCAAAGGGAATGTG) expression using Pfaffl method [34].

### Immunoblotting

For immunoblotting, the cells from 2D culture were harvested in RIPA sample buffer (containing 150 mM TRIS-HCl, 1% NP-40, 150 mM NaCl, 0.5% Nadeoxycholate, 1 mM EDTA, 1 mM SDS). For 3D culture lysates, the organoids were first harvested from Matrigel as described for RNA isolation and combined from several cultures in one pellet. The pellet was lysed in buffer containing 20 mM TRIS-HCl, 1% Triton X100, 100 mM NaCl, 1 mM EDTA and 1× protease and phosphatase inhibitors (A32955 and A32957, Pierce, Thermo Fisher). SDS sample buffer (containing 60% Glycerol, 360 mM Tris pH 6.8, 12% SDS, 0,06% Bromophenolblue, 6.6% β-mercaptoethanol) was added to the lysates. Proteins were separated on BioRad Mini-Protean TGX 4–20% gels (Cat. #456-1094) and transferred to PVDF-membrane using BioRad’s SemiDry system with 25 V for 30 min. Membranes were blocked with 3% BSA 5% fat-free milk solution. Immunoblot analyzes were performed using rabbit anti-AML1 (1:1000), rabbit anti-RUNX2 (1:1000), rabbit anti-Phospho-MEK1/2 (1:1000), rabbit anti-MEK1 (1:1000), rabbit anti-Phospho-p44/42 MAPK (Erk1/2) (1:1000), rabbit anti-p44/42 MAPK (Erk1/2) (1:1000), rabbit anti-NOTCH1 (1:1000), rabbit anti-NOTCH2 (1:1000), rabbit anti-HES1 (1:1000), rabbit anti-HES5 (1:1000) or mouse anti-β-actin (1:2500) antibodies at + 4 °C overnight and then 1 h at RT with horseradish peroxidase-conjugated secondary antibodies anti-rabbit IgG (1:5000) or anti-mouse IgG (1:5000) and chemiluminescence detection reagents from PerkinElmer (Cat. NEL122001EA).

Chemiluminescence signals were visualized with Fujifilm LAS 4000 gel imager and the signal intensities were quantified from JPEG images with ImageJ software as instructed by Davarinejad H. (http://www.yorku.ca/yisheng/Internal/Protocols/ImageJ.pdf. Accessed January 6, 2023.) Briefly, inverted specific protein signal intensity values (PSI) were measured and background was subtracted. The PSIs from proteins of interests were normalized to the total protein loading control PSIs and presented as relative values. For phospho-proteins, the PSIs of the phospho-proteins and corresponding total proteins were first normalized to the global loading control and then the normalized phospho-protein PSI was further normalized to the corresponding total protein normalized PSI. The intensities are shown relative to the Ctr samples.

### Fluorescence staining of 3D cultures, imaging and quantitative morphometric analysis

At the endpoint of an experiment, the organotypic 3D cultures were always first imaged with wide-field phase-contrast microscope (Zeiss Axiovert 200 M with AxioCam MRm camera). For phalloidin staining, the 3D cultures on 96-well angiogenesis µ-plates were fixed and permeabilized in 2% paraformaldehyde-0.5% Triton-X100 in PBS (20 min, + 37 °C) and stained with Alexa Fluor™ 546 phalloidin 1:200, 1 h at RT (Thermo Fisher Scientific). Hoechst33342 was used as a nuclear counter stain. For immunofluorescent staining, the organoid-like structures were harvested from Matrigel as described above for RNA isolation, fixed in 2% paraformaldehyde, casted in Histogel (Thermo Scientific) and embedded in paraffin for sectioning. The sections were rehydrated and antigen retrieval was performed in a pressure cooker for 20 min in 10 mM citrate buffer (pH 6.0). Blocking against non-specific antibody binding was done with 10% BSA in PBS-0.1% Tween for 1 h at RT. Primary antibody incubations with anti-Vimentin (Cell Signaling Technologies, cat. #5741, 1:100) were carried out at 4 ºC for overnight in the blocking solution. The samples were then incubated with AlexaFluor594 secondary antibody (1:500) in blocking solution for 1 h at RT. After washing, all the sections were mounted with ProLong Diamond Antifade Mountant with DAPI for nuclear staining (cat. # P36962, Thermo Fisher Scientific). The stainings were imaged with 3i CSU-W1 spinning disk confocal microscope using 40× LD objective.

For quantitative morphometric analysis, the organoid-like structures were stained with live cell dyes Calcein-AM and Ethidiumhomodimer-2 (EthD2) (both from Thermo Fisher Scientific) to visualize living and dead cells, respectively. The structures were then imaged with a spinning disk confocal microscope (Axiovert 200 M, 5× objective) and the image stacks were converted to maximum intensity projections with SlideBook6 software (3i Intelligent Imaging Innovations Inc.). The image projections were analyzed using an automated morphometric image data analysis software AMIDA [23]. The resulting data derived from the 3D culture was further visualized and used for comparisons with the R-software environment (www.r-project.org).

### Quantification and statistical analysis

The statistical analyzes were done with the GraphPad Prism 9 software (GraphPad Software, La Jolla, CA, USA) if not mentioned otherwise. Significance was determined as *p ≤ 0.05, **p ≤ 0.01, ***p ≤ 0.001.

For Fig. 3C, the statistical differences in cell adhesion were determined by two-way ANOVA for repeated measures and Tukey’s multiple comparison test.

For Fig. 3D, 2D proliferation was determined by nuclear counting over time, and normalized to 6 h timepoint in the corresponding well, with IncuCyte S3 software (2020A). Data were analyzed by calculating the areas under curve (AUC) for the growth curves and comparing the AUC values against the control (Ctr) with ordinary one-way ANOVA, along with Dunnett’s multiple comparisons test.

For Fig. 3G, morphometric data derived from cell organoids was received with AMIDA software. *Roundness* describes how round an individual object is and *AppIndex* describes the severity of cellular protrusions reaching out from the object body. Data visualization and statistical analysis were performed in the R-software environment (www.r-project.org) using Bonferroni-corrected t tests against Ctr, 12 wells/treatment.

For Fig. 3J, relative wound density over time was determined with IncuCyte S3 software (2020A). Data were analyzed by calculating the areas under curve (AUC) and comparing the AUC values with unpaired t test.

For Fig. 3K, the data for 3D cell numbers were normalized to the values from Day 1 (16 h after seeding), and the values derived from the wells analyzed at different timepoints were compared via multiple t-tests along with Holm-Šídák multiple comparisons test.

For Fig. 4D, the statistical testing between sample groups was carried out using the DESeq2 R package (2_1.6.3). The resulting p values were adjusted using the Benjamini and Hochberg’s approach for controlling the false discovery rate (FDR).

For Fig. 4E, statistical analysis was performed with unpaired t test (per row w/ individual variances) and Holm-Šídák test was used for multiple comparisons.

For Fig. 4F, *AppIndex* morphometric data of cell organoids were received with AMIDA software. *AppIndex* describes the severity of cellular protrusions reaching out from the object body. Data visualization and statistical analysis were performed in the R-software environment (www.r-project.org) using Bonferroni-corrected t tests against untreated Ctr and ddR1 + R2 cultures, 6 wells/treatment.

## Results

### Gene expression is altered in the Dicer1 cKO epididymis

**Fig. 1:**
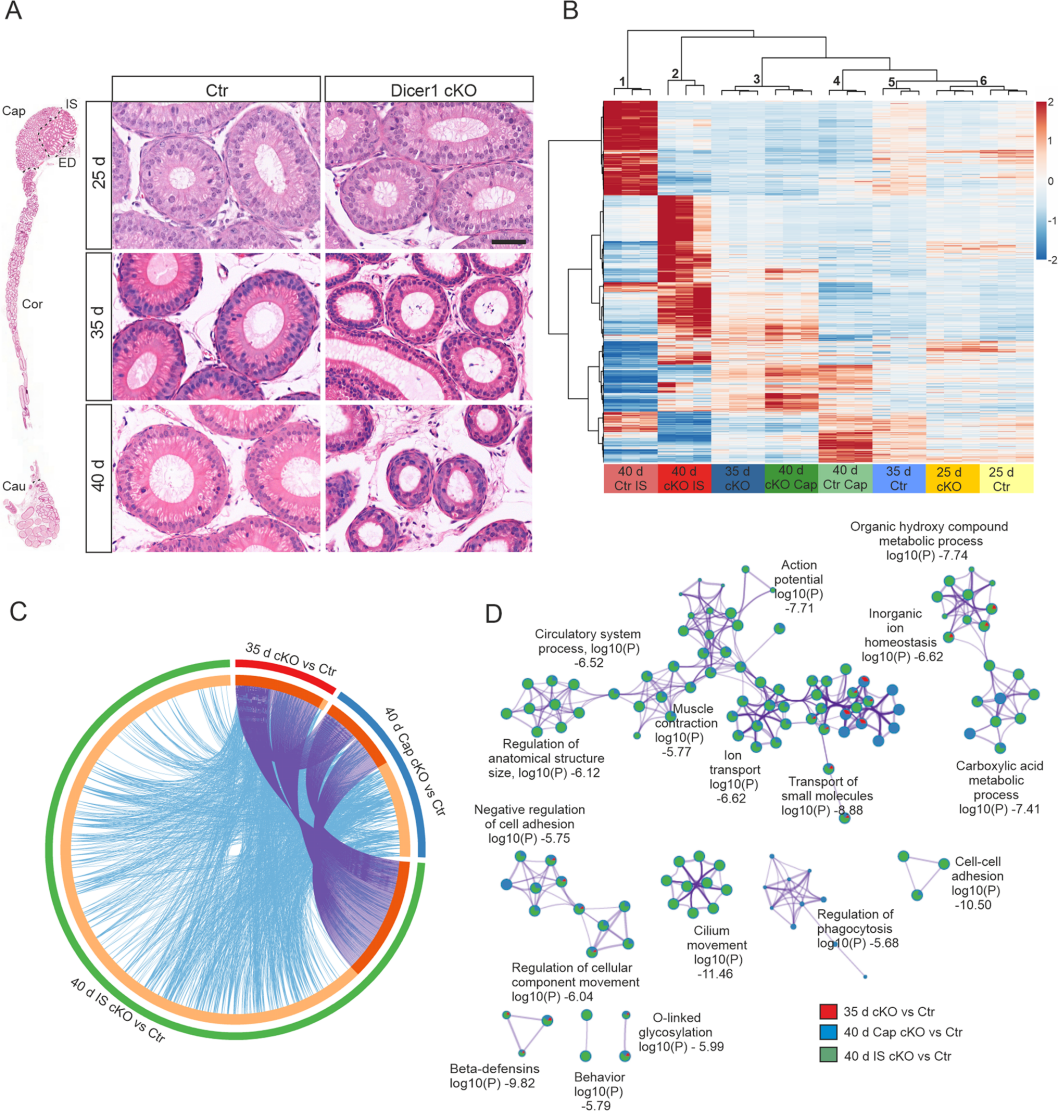
Lack of Dicer1 disrupts epididymal epithelium differentiation and gene expression. **A** Hematoxylin and eosin stained section depicting the whole epididymis of 40-day-old control (Ctr) mouse with different segments marked. Whole epididymides were used for RNA-seq at 25 and 35 days, whereas separated initial segment (IS) and Caput (Cap), were used at 40 days of age. ED, efferent ducts; Cor, corpus; Cau, cauda. Higher magnification sections of Ctr and Dicer1 cKO mouse initial segment at the age of 25, 35 and 40 days. Scale bar 20 µm. **B** Heat map of deregulated genes clustered by unsupervised hierarchical clustering. RNA-seq data from Dicer1 cKO and Ctr proximal epididymis at the ages of 25, 35 and 40 days. **C** Circos plot from differentially expressed (DE) genes. Purple curves link identical genes and blue curves link the genes enriched in the same ontology term. The inner circle represents DE gene lists, where hits are arranged along the arc. Genes that hit multiple lists are colored in dark orange, and genes unique to a list are shown in light orange. **D** The network of the enriched GO terms in the top 17 DE pathways. The GO terms are presented as pie charts, where the size of a pie is proportional to the total number of hits that fall into that specific term. The pie charts are color-coded based on the gene list identities, where the size of a slice represents the percentage of genes under the term that originated from the corresponding gene list

We previously demonstrated that the proximal epididymal epithelium of Dicer1 cKO mice begins to differentiate and appears normal at the age of 33 days. However, at the age of 45 days, the epithelium, especially at IS, had started to regress back to an undifferentiated state [17]. To obtain a more comprehensive view of the timeline of the loss of proper epithelial differentiation, we further analyzed the epididymal histology at three additional age points; 25, 35 and 40 days of age. At the age of 25 days, the Dicer1 cKO epithelium appeared histologically normal, whereas at 35 days, the regression of the epithelium had started, judged by the epithelial cell height. At the age of 40 days, the histology clearly represented an undifferentiated epithelium (Fig. 1A).

In order to identify the factors behind the observed loss of normal epithelial differentiation state, we performed RNA-seq on the proximal epididymis at the above-mentioned ages (Table S2). Corresponding to the histological appearance of the samples, the unsupervised hierarchical clustering of deregulated genes placed the 25 days control (Ctr) and 25 days Dicer1 cKO samples in the same cluster (cluster 6, Fig. 1B). The 35 days Ctr and 40 days Ctr Cap samples were grouped in the adjacent clusters 5 and 4, respectively, whereas the 35 days Dicer1 cKO samples along with the 40 days Dicer1 cKO Cap samples clustered together into cluster 3. The 40 days IS samples from Ctr and Dicer1 cKO animals clustered separately into clusters 1 and 2, respectively (Fig. 1B). As expected, at the age of 25 days the gene expression patterns in Dicer1 cKO and Ctr epididymides showed high similarity and no genes were identified to be differentially expressed (DE) with the used strict criteria, fold change (FC) 2 and False Discovery Rate (FDR) 0.001. However, with less strict criteria, FC2 and FDR 0.05, altogether 38 genes were found changed (12 up- and 26 down-regulated). At the age of 35 days, altogether 189 DE genes were identified (72 up, 117 down), and at 40 days of age, the number of DE genes was 1558 in IS (934 up, 624 down) and 346 in caput (222 up, 124 down) in Dicer1 cKO samples when compared to Ctr (FC2 and FDR 0.001). Altogether 70 DE genes were shared when comparing the 35-day proximal epididymis and the 40-day IS and caput (Dicer1 cKO vs Ctr). In addition, 99 DE genes were shared between the 35 days and 40 days IS comparisons, whereas the 35 days and 40 days Cap comparisons shared only 10 DE genes (Fig. 1C) corresponding to histological analyzes, where the changes started to appear at the age of 35 days and got more pronounced by the age of 40 days particularly in IS. The top pathways associated with DE genes at different age points contained for example ion and small molecule transport, cell adhesion, regulation of anatomical structure size and metabolic processes of organic hydroxy compounds and carboxylic acid (Fig. 1D). In the Dicer1 cKO mice, the efferent ducts are highly enlarged [17] and GO term cilium movement among the top DE pathways suggest that the initial segment samples may have contained small pieces of efferent ducts.

Tight junctions maintain the barrier function and apical-basolateral polarity in the differentiated epithelium. To further analyze the changes related to cell adhesion and differentiation in the Dicer1 cKO epididymis, we performed immunofluorescent analysis of several tight junction proteins (TJPs); TJP1, TJP2 and TJP3, and claudins (CLDN); CLDN1, CLDN3 and CLD4 in adult 2-month-old Ctr and Dicer1 cKO epididymides. In Dicer1 cKO epididymides the expression of TJP1 and TJP2 was markedly reduced and discontinuous, whereas TJP3 seemed to be upregulated in Dicer1 cKO, but with similarly discontinuous pattern (Fig. S2). Moreover, claudins were clearly delocalized (Fig. S2), and along with TJP results, suggested severe functional defects in tight junction formation.

### The expression of TFs in developing mouse epididymis

**Fig. 2:**
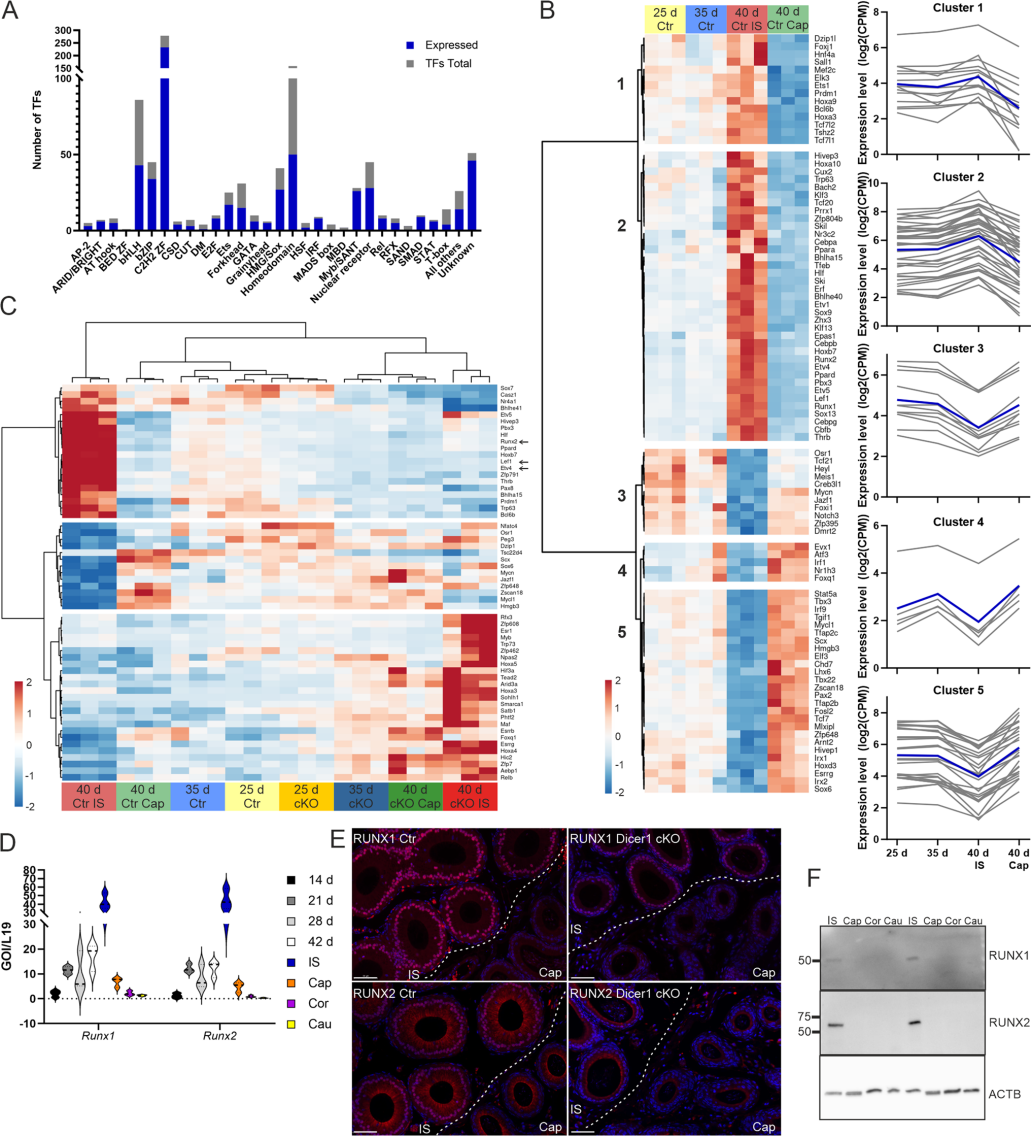
Transcription factors expressed in the Ctr and Dicer cKO epididymides. **A** Number of TFs identified in each TF family, out of all family members, in the Ctr mouse epididymis in any of the ages sequenced. The TFs were classified into families according to their DNA-binding domains. **B** Heat map with subcluster plots from differentially expressed TFs between ages analyzed from Ctr epididymis. **C** Heat map of DE TFs between Ctr and Dicer1 cKO epididymides at different ages. *Runx2*, *Lef1* and *Etv4* are highlighted with arrows. **D** Violin plots of *Runx1* and *Runx2* mRNAs from the wild type (WT) mouse epididymis at different ages and from 2-month-old adult epididymal segments. In the plots dashed line represents median, dotted lines first and third quartile. Zero-level is high-lighted with a black dotted line. **E** Immunofluorescent localization of RUNX1 and RUNX2 in the IS/Cap epididymis in Ctr and Dicer1 cKO mice at the age of 40 days. RUNX1 and RUNX2, red; DNA, blue. Scale bar 50 μm. **F** Immunoblotting of RUNX1 and RUNX2 in adult 2-month-old WT epididymis. Immunoblotting of beta-actin (ACTB) levels was used to control protein loading. *IS* initial segment, *Cap* caput, *Cor* corpus, *Cau* cauda

As TFs drive gene expression programs behind development and maintenance of tissue types, we next analyzed changes in TF expression in the Dicer1 cKO epididymides. Among the 952 analyzed mouse TFs, 625 were expressed in the Ctr epididymis (Fig. 2A), and 93 TFs demonstrated changes in expression levels during segment differentiation (Fig. 2B). Clusters 1 and 2 contain TFs expressed mainly in the developing IS, whereas clusters 3–5 include TFs with higher expression in developing caput compared to IS (Fig. 2B). Fifteen TFs showed fragments per kilobase per million mapped fragments (FKPM, RNA-seq) > 100 in the proximal epididymis in at least one of the ages studied (Table 1). The majority of these were expressed at high level in all samples, except E74-like factor 3 (*Elf3*) and transcription factor 7, T cell specific (*Tcf7*) which were highly expressed in the 40-day Cap samples, while early growth response 2 (*Egr2*), ets variant 4 and -5 (*Etv4*, -*5*) and homeobox B7 (*Hoxb7*) showed high expression in the IS, suggesting a segment-specific role for these TFs. Furthermore, when compared to the human TF Atlas [31], four out of the 15 TFs are reported highly expressed also in the human epididymis (Table 1).

**Table 1:** Transcription factors with highest expression level in the mouse proximal epididymis

MGI symbol	DBD	25d Ctr (FPKM)	35d Ctr (FPKM)	40d Ctr IS (FPKM)	40d Ctr Cap (FPKM)	Human epididymis^a^
*Atf4*	bZIP	122.8	147.5	175.4	147.0	Low exp. (TPM < 10)
*Drap1*	Unknown	110.2	129.6	102.7	121.7	Low exp. (TPM < 10)
*Egr2*	C2H2 ZF	168.2	163.1	255.4	132.8	Intermediate exp. (TPM 10–50)
*Elf3*	Ets	82.3	81.8	24.6	122.8	Low exp. (TPM < 10)
*Etv4*	Ets	42.3	48.2	134.8	6.9	Low exp. (TPM < 10)
*Etv5*	Ets	61.1	72.9	192.7	16.0	Low exp. (TPM < 10)
*Hoxb6*	Homeodomain	132.8	160.4	216.3	145.2	High exp. (TPM > 50)
*Hoxb7*	Homeodomain	96.5	114.1	184.7	91.7	High exp. (TPM > 50)
*Hoxb8*	Homeodomain	89.2	104.8	133.9	100.4	High exp. (TPM > 50)
*Hoxd4*	Homeodomain	126.7	107.7	89.1	121.1	Intermediate exp. (TPM 10–50)
*Hoxd8*	Homeodomain	107.4	109.8	105.4	122.5	High exp. (TPM > 50)
*Pax8*	Prd/Homeodom	91.5	97.8	145.4	86.0	Intermediate exp. (TPM 10–50)
*Srebf1*	bHLH	105.3	100.1	123.7	89.9	Low exp. (TPM < 10)
*Tcf7*	HMG/Sox	76.4	83.2	25.4	147.0	Intermediate exp. (TPM 10–50)
*Xbp1*	bZIP	108.0	120.6	140.6	123.7	Low exp. (TPM < 10)

*DBD* DNA binding domain, *FPKM* Fragments Per Kilobase per Million mapped fragments, *TPM* transcript per million

^a^Lambert et al., 2018 Cell [21]

From all the TFs expressed in the mouse epididymis, 58 were dysregulated in Dicer1 cKO epididymides (Fig. 2C). Interestingly, only three TFs showed expression patterns associated with observed histological differences. *Runx2*, lymphoid enhancer binding factor 1 (*Lef1*) and *Etv4* were expressed in the 25 days Dicer1 cKO epididymis at a comparable level to Ctr but were significantly down-regulated by the age of 35 days and remained down-regulated in the IS of the 40-day-old Dicer1 cKO epididymides as compared to Ctr (Fig. 2C arrows and Table S2). Furthermore, *Runx1* was significantly downregulated 40-day-old Dicer1 cKO IS (log2FC − 0.7, adj.p ≤ 0.001). In the caput, the expression levels of *Runx2, Lef1* and *Etv4* were very low with no change between the genotypes. Full knock-out of *Etv4* does not cause changes in the epididymal histology [35], whereas Lef1 knockout mice die postnatally before weaning with macroscopically normal urogenital tract [36]. Interestingly, *Runx* transcription factors are missing from two other mouse models, c-*ros* knock-out and transgenic GPX5-Tag2, which both lack functional IS [37]. Thus, we decided to study the role of RUNX TFs in epididymal development in more detail.

From the three mammalian RUNX family members, *Runx1* and *Runx2* were detected in the Ctr epididymis by RNA-seq. A more detailed analysis from WT epididymis with RT-qPCR revealed mRNA expression of both family members in the proximal epididymis at the age of 14 days, from where the expression levels increase, being highest in the adult IS. Low levels of *Runx1* and *Runx2* mRNA were also detected in other adult epididymal segments (Fig. 2D). Immunofluorescent staining at the age of 40 days revealed nuclear expression of RUNX1 and RUNX2 in the IS epithelial cells in Ctr mice, but not in the caput. In Dicer1 cKO epididymides, RUNX1 and -2 showed markedly reduced immunostaining (Fig. 2E), which was in accordance with the RNA-seq data. In both analyses, RUNX2 down-regulation was more pronounced. Using immunoblotting, RUNX1 and RUNX2 proteins were observed exclusively in the IS of WT epididymis (Fig. 2F).

### ***Generation of epididymal epithelial cell lines with runx1 and ***−***2 deletions***

In order to study the role of RUNX TFs in the epididymal epithelium, we used CRISPR-Cas9 genome editing to generate mE-Cap18 cell lines lacking functional RUNX1, RUNX2 or both. To inactivate RUNX function, we aimed to delete the essential exon coding Runt-domain, responsible for DNA-binding and protein-protein interaction, located at exon 5 in *Runx1* and exon 4 in *Runx2* (Fig. S1). The generated cell lines will be referred herein as dR1, dR2 and ddR1 + R2, whereas cells treated with negative control crRNA and untreated cells will be referred as Ctr and WT cells, respectively.

**Fig. 3:**
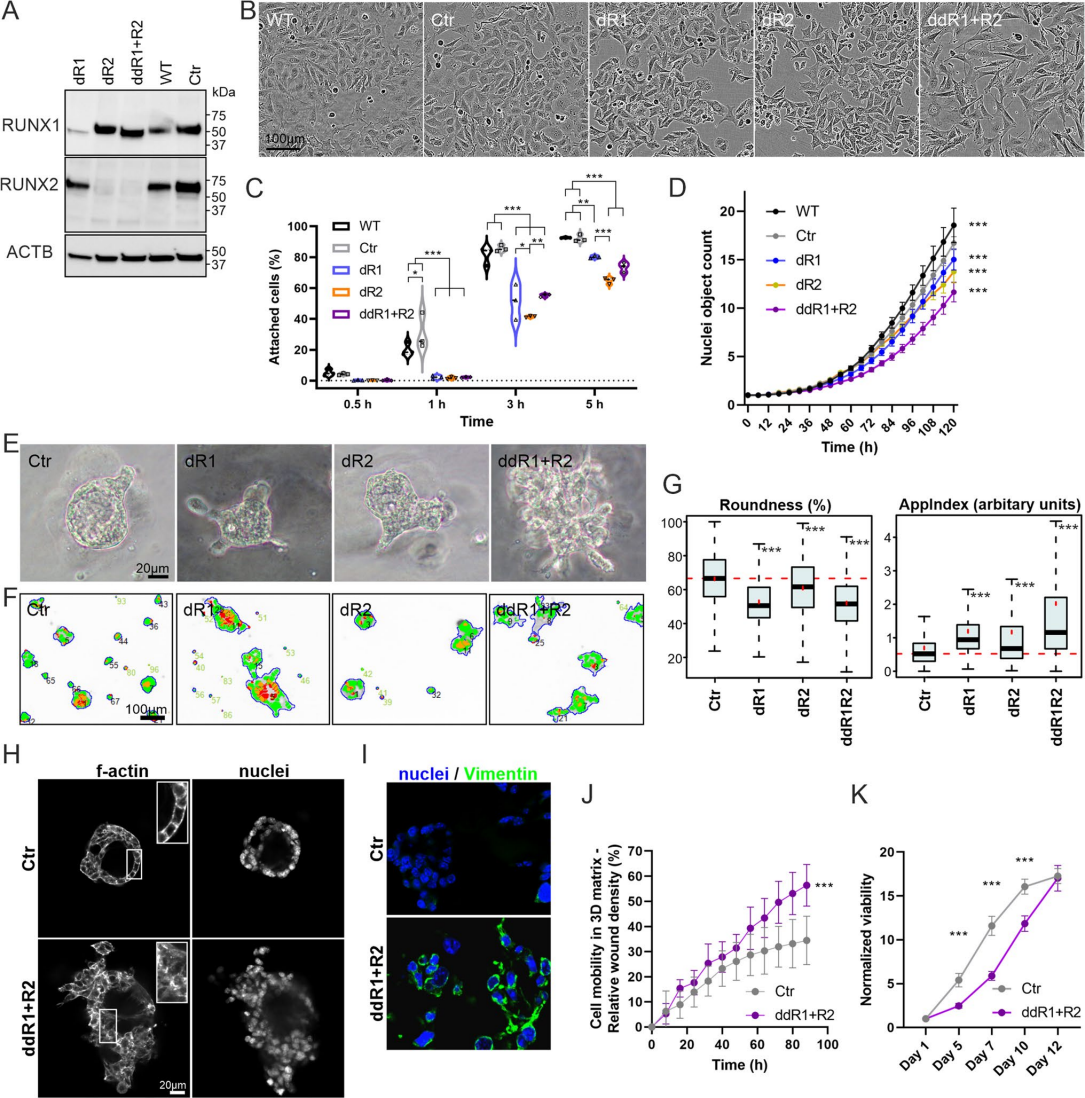
RUNX functional deletion affects the mouse epididymal epithelial cell phenotype in vitro. **A** Immunoblot for RUNX1 and RUNX2 proteins in lysates from mouse epididymal epithelial cells harboring mutations in DNA-binding domain of *Runx1* and *Runx2* genes. Beta-actin (ACTB) was used as a loading control. **B** Cell morphology in 2D cultures imaged 3 days after plating. **C** Quantification of attached cells at indicated time points. *p ≤ 0.05, **p ≤ 0.01, ***p ≤ 0.001, n = 3, appr. 500 cells/replicate. **D** Cell proliferation rate in 2D culture based on nuclear counts using NucLight Red and IncuCyte S3 real-time imaging. The normalized counts are shown on y-axis (normalized to 6 h). Results are presented as mean and SD. ***p ≤ 0.001, n = 12. **E** Phasecontrast images from organoids in 3D Matrigel cultures at day 10 (Ibidi-plates). **F** Examples of AMIDA segmentation generated from confocal microscope images of organoids at day 10 of culture. **G** *Roundness* and *AppIndex* measures from morphometric data presented as a box and whisker plot (median, black line; mean, red spot). ***p ≤ 0.001, 12 wells/cell line. **H** Confocal images from organoids in Matrigel at day 12. The cultures were fixed and stained with phalloidin (f-actin) and Hoechst33342 (DNA/nuclei). **I** Immunofluorescent staining of vimentin (green) in paraffin sections of Ctr and ddR1 + R2 organoids (day 12, 3D culture in Matrigel). Hoechst33342 (blue) was used as a counterstain for DNA/nuclei. **J** Quantitative analysis of cell movement inside 3D Matrigel (invasion) with Scratch wound assay and IncuCyte S3. Results are presented as means and SD. ***p ≤ 0.001, Ctr n = 7, ddR1 + R2 n = 9. **K** Quantification of living cells by metabolic activity measurement with WST8 (CCK-8 kit) in organotypic 3D cultures in five timepoints over the 12 days culturing period. Results are presented as mean and SD. ***p ≤ 0.001, n = 6

The effects of deletions on *Runx1* and *Runx2* mRNA expression was analyzed using RT-qPCR. The dR1 cell line showed a gene expression ratio of 0.7E−04 for *Runx1* and 1.0 for *Runx2* compared to parental cells. The ratios observed for the dR2 cell line were 1.0 for *Runx1* and 1.5E−04 for *Runx2*. For the ddR1 + R2 cell line, the observed ratios were 3.1E−04 for *Runx1* and 0.9 for *Runx2.* The deletion of exon 5 of *Runx1* is expected to result in a truncated protein lacking 35 amino acids in dR1 and ddR1 + R2 cell lines. Immunoblot analysis produced a smaller RUNX1 band in dR1 and ddR1 + R2 cell lines corresponding to a truncated protein (Fig. 3A). In contrast, the different *Runx2* deletions appeared to result in an almost complete loss of RUNX2 protein in both dR2 and ddR1 + R2 cell lines (Fig. 3A).

### RUNX1 and RUNX2 knockout affects epididymal cell phenotype in vitro

On a plastic surface, non-malignant epithelial cells typically grow as confluent monolayers. The cell shape is generally polygonal with well-defined boundaries. The nucleus of an epithelial cell often is round or oval in shape and has clearly visible nucleoli. While the WT and Ctr cells exhibited many characteristics of normal epithelial cells on the 2D cell culture, ddR1 + R2 cells clearly showed a more mesenchymal appearance (Fig. 3B). This was demonstrated by the elongated and often bipolar shape with long cell extensions and poorly detectable cell-cell-contacts. ddR1 + R2 cells were also growing as single cells rather than a continuous cell layer (Fig. 3B). Some morphological changes were also detected in dR1 and dR2 cells but in contrast to ddR1 + R2 cells, the overall epithelial phenotype was retained (Fig. 3B). Many of the observed characteristics of ddR1 + R2 cells are typical for mesenchymal cells and linked to epithelial to mesenchymal transition (EMT) [38]. Furthermore, we noted that the cell attachment to the culture substratum after plating was significantly delayed in dR1, dR2 and ddR1 + R2 cells when compared to WT and Ctr cells (Fig. 3C) indicating alterations in cell adhesion. In 2D cultures, the cell proliferation rate was reduced in ddR1 + R2 cells, and in a smaller degree also in dR2 and dR1 cells, compared to either of the control cell lines WT and Ctr (Fig. 3D). However, the delay in cell attachment may have impacted this result.

As RUNX1 and -2 deficient epididymal cells partially demonstrated less typical epithelial morphologies compared to control cells in a conventional 2D cell culture, we next sought to assess the ability of these cells to form well-defined, self-assembled organoid-like structures in 3D Matrigel matrix. Potentially, these structures recapitulate some characteristics of differentiated epithelium, such as acini or ducts with intact cell-cell contacts, even when generated from cell lines [39]. From here on, these organoid-like structures will be called organoids for simplicity. In these organotypic 3D cultures, Ctr cells formed mainly round and small organoids, occasionally with a clear hollow lumen inside, while epididymal cells with ddR1 + R2 deletion failed entirely to form organized structures, suggesting a significantly reduced capability for epithelial differentiation (Figs. 3E and S3). While the organoid surface was smooth in Ctr, dR1 and dR2 organoids, the ddR1 + R2 organoids were highly irregular and lacked clear boundaries with individual cells reaching out from the organoid body in a very unorganized manner. Occasional hollow structures detected in ddR1 + R2 organoids were not well defined. The roundness of organoids was reduced and the severity of “invasive”-like extensions (*AppIndex*) increased significantly in dR1, dR2 and ddR1 + R2 cells when compared to Ctr cells showing the greatest difference to ddR1 + R2, as detected with the AMIDA phenotypic image analysis (Fig. 3F–G).

Furthermore, demonstrating the striking cytoskeletal remodeling observed in ddR1 + R2 organoids, staining of actin filaments (F-actin) revealed a severely disorganized cortical actin cytoskeleton, suggesting disrupted intercellular contacts and severely reduced epithelial integrity, compared to Ctr cells (Fig. 3H). Moreover, immunostaining of paraffin-embedded sections of organoids showed that ddR1 + R2 cells express marked levels of vimentin (Fig. 3I). These observations further imply that at least partial EMT has taken place in ddR1 + R2 cells. To examine motile activity of ddR1 + R2 cells, we performed a scratch wound assay in 3D Matrigel matrix. Here, ddR1 + R2 cells showed significantly enhanced mobility and wound closure compared to Ctr cells (Fig. 3J). Finally, we found that cell proliferation rate was similar in Ctr cells and ddR1 + R2 cells after reaching the exponential growth phase in organotypic cultures, as detected with cell viability assay. However, Ctr cells seemed to stabilize faster to the 3D culture environment and thus initiation of exponential growth was delayed in ddR1 + R2 cells (Fig. 3K). ddR1 + R2 cells and especially dR1 cells formed significantly bigger organoid-like structures than Ctr cells (*Area*, Fig. S4). As cell viability per well for Ctr and ddR1 + R2 cells remained the same at the assay end point, the size difference may be due to a certain level of organoids or cell clusters merging together. Indeed, the number of ddR1 + R2 and dR1 organoids detected at the experiment endpoint was less than Ctr (Fig. S4).

### Lack of functional RUNXs affects MAPK signaling

**Fig. 4:**
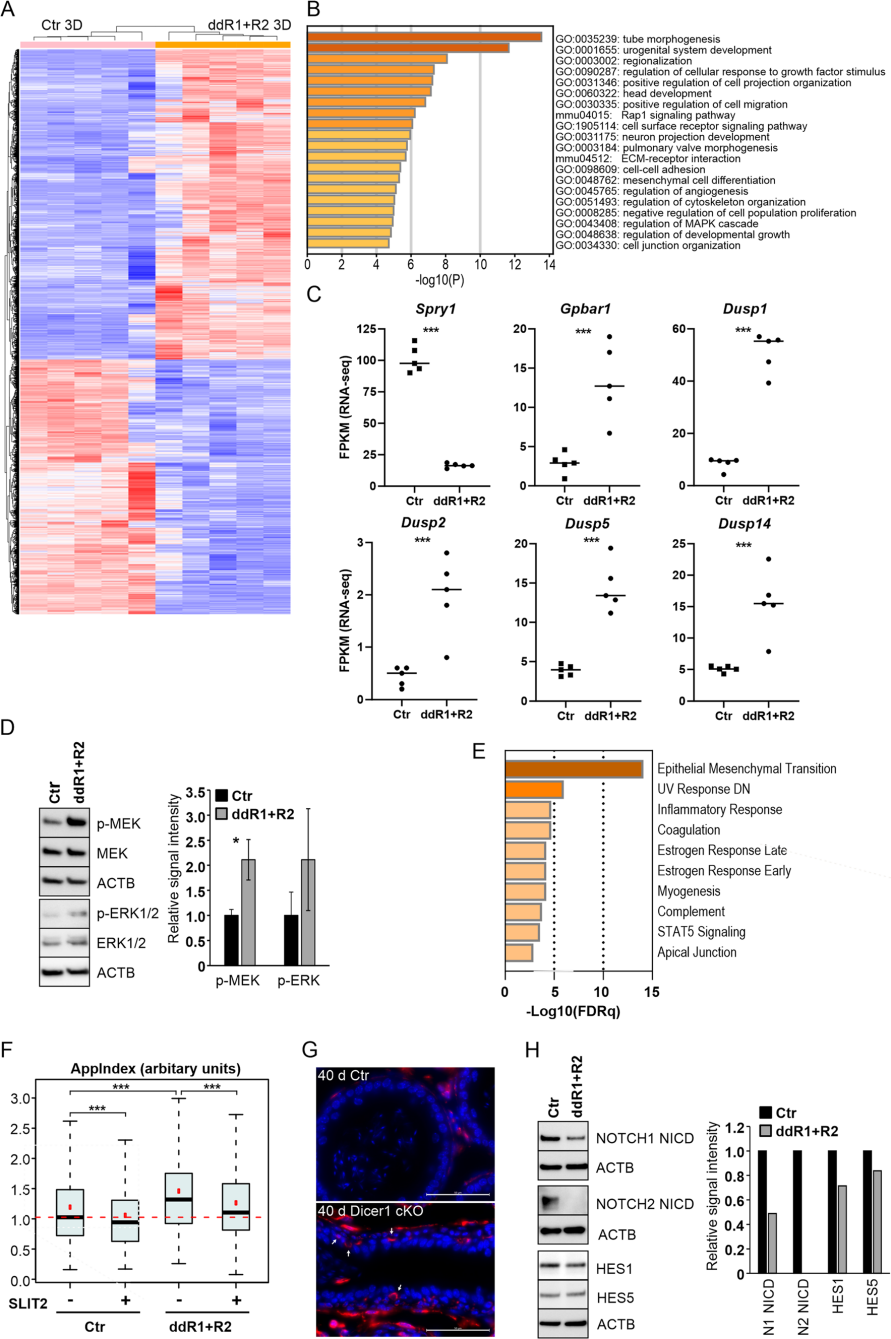
Lack of RUNX1 and RUNX2 affects several signaling pathways in organotypic cultures. **A** Heat map of DE genes clustered by unsupervised hierarchical clustering. The data present RNA-seq of Ctr and ddR1 + R2 organoids cultured in 3D Matrigel for 12 days. See also Fig. S3. **B** Top twenty GO terms associated with down-regulated DE genes in ddR1 + R2 organoids. **C** RNA-seq data of MAPK-pathway regulators sprouty RTK signaling antagonist 1 (*Spry1*), G protein-coupled bile acid receptor 1 (*Gpbar1*), dual specificity phosphatase (*Dusp*) family members 1, 2, 5 and 14. ***p ≤ 0.001. FPKM, fragments per Kb of exon per million fragments mapped. **D** Representative immunoblot analysis of total and phosphorylated MEK and ERK1/2 in Ctr and ddR1 + R2 cells. Immunoblotting of beta-actin (ACTB) levels was used to control protein loading. Quantification was done from three independent experiments. *p ≤ 0.05. **E** MSigDB Hallmark gene set analysis for down-regulated DE genes (log2FC < − 1.5, p ≤ 0.05) in 3D ddR1 + R2 organoids. FDRq is a false discovery rate analog of hypergeometric p value after correction for multiple hypothesis testing according to Benjamini and Hochberg. **F** *AppIndex* morphometric measure of Ctr and ddR1 + R2 organoids cultured in 3D Matrigel with or without recombinant slit guidance ligand 2 (SLIT2, 0.5 µg/ml). ***p ≤ 0.001, 6 wells/test condition.** G** Immunofluorescent staining of vimentin (VIM) in 40-day-old control (Ctr) and Dicer1 cKO epididymides. VIM red; DNA, blue. Scale bars 50 μm. **H** Immunoblot analysis of NOTCH intracellular domains (NICD) of NOTCH1 and NOTCH2 receptors, hes family bHLH transcription factor 1 (HES1) and hes family bHLH transcription factor 5 (HES5) in organoids cultured for 12 days. Beta-actin (ACTB) levels were used to control protein loading

To elucidate the molecular mechanisms behind the observed phenotype in ddR1 + R2 cells, we next examined transcriptomic changes in ddR1 + R2 organoids by RNA-seq analysis of Ctr and ddR1 + R2 organoids harvested from 3D Matrigel matrix. Altogether 3109 genes were down-regulated whereas 3787 genes up-regulated in ddR1 + R2 organoids compared to Ctr (Fig. 4A and Table S2) (adj.p ≤ 0.05). The DE genes included *Vim* (log2FC 0.8, adj.p ≤ 1.8E−08) correlating with observed up-regulation of protein levels. In addition, several known RUNX target genes, such as Vav3 oncogene (*Vav3*, log2FC − 1.2, adj.p ≤ 1.9E−10) [40] and integrin beta 3 (*Itgb3,* log2FC −4.3, adj.p ≤ 6.5E−36) [41] were down-regulated, thus further validating the model used. Interestingly, the top GO terms associated with the down-regulated genes included for example: *tube morphogenesis*, which is analogical with features of epithelia differentiation, *cell-cell adhesion* and *regulation of MAPK cascade* (Fig. 4B). Furthermore, GO terms *positive regulation of cell projection organization* and *positive regulation of cell migration* were adduced by the pathway analysis of down-regulated genes, showing a connection with the phenotypic alterations observed in ddR1 + R2 organoids (Fig. 3E, G, I). The top pathways associated with the up-regulated DE genes included for example: *transport of small molecules* and *anion transport* (Fig. S5). From the RNA-seq data, we noted that the expression of several MAPK-pathway regulators was markedly changed in ddR1 + R2 cells. Sprouty RTK signaling antagonist 1 (*Spry1*), a known upstream inhibitor of MAPK signaling [42], was significantly down-regulated in ddR1 + R2 organoids (log2FC − 2.6, adj.p ≤ 2.7E−80), whereas an upstream activator of MAPK-signaling, G protein-coupled bile acid receptor 1 (*Gpbar1*), was significantly up-regulated (log2FC 2.2, adj.p ≤ 8.2E−08) (Fig. 4C), suggesting over activation of MAPK signaling. Furthermore, several dual specificity phosphatase family members (DUSPs), targeting MAP-kinases such as ERK1/2 [43], were up-regulated, including *Dusp1* (log2FC 2.6, adj.p ≤ 6.0E−27), *Dusp2* (log2FC 2.1, adj.p ≤ 1.4E−06), *Dusp4* (log2FC 0.6, adj.p ≤ 0.05), *Dusp5* (log2FC 1.9, adj.p ≤ 6.2E−19), *Dusp6* (log2FC 1.4, adj.p ≤ 0.005), *Dusp7* (log2FC 0.6, adj.p ≤ 0.005) and *Dusp14* (log2FC 1.6, adj.p ≤ 2.4E−09) (Fig. 4C). These results, along with previous data showing that IS differentiation is governed by MAPK signaling [7], prompted us to analyze the activity of the MAPK pathway in ddR1 + R2 cells at protein level. Interestingly, we found that the phospho-MEK levels were indeed significantly increased in ddR1 + R2 cells compared to Ctr (Fig. 4D). Furthermore, downstream in the signaling cascade, the levels of phospho-ERK1/2 were also increased although due to experimental variation, did not reach statistical significance (Fig. 4D). Thus, our data suggests that RUNXs participate in the regulation of MAPK signaling pathway activity in the epididymal epithelial cells.

### Lack of functional RUNX1 and RUNX2 is associated with changes in EMT-related gene expression

To further elaborate the transcriptomic analysis, we searched the MSigDB database for the potential enrichment of different Hallmark gene sets in our RNA-seq data from ddR1 + R2 organoids. Consistent with our previous phenotypic observations pointing towards partial EMT, namely disorganized organoid formation, changes in cytoskeletal actin and vimentin, and increased mobility by ddR1 + R2 cells, the DE genes were enriched most significantly in the hallmark *Epithelial to mesenchymal transition* (FDRq = 1.6 e^−15^) with 20 down-regulated genes (Fig. 4E). One of the EMT-related genes down-regulated in ddR1 + R2 organoids was slit guidance ligand 2 (*Slit2*, log2FC − 10.7, adj.p ≤ 2.1E−27). As SLIT2 has been shown to inhibit cell migration of colorectal cancer cells [44], we wanted to analyze whether supplementing ddR1 + R2 cell cultures with SLIT2 recombinant protein would affect the enhanced mobility of the ddR1 + R2 cells. However, we did not detect any effects on cell movement in a scratch wound model in 3D Matrigel matrix (data not shown). Instead, SLIT2 significantly decreased the severity of the cellular protrusions in the ddR1 + R2 organoids (*AppIndex*), which typically is a measure of collective cell invasion and branching in organotypic 3D culture (Fig. 4F). To further analyze the cellular identity of ddR1 + R2 cells, we examined the expression of several epithelial keratins in RNA-seq data from the Ctr and ddR1 + R2 cells in vitro and the Ctr epididymal tissue (Table 2). Epithelial keratins *Krt10*, *Krt18* and *Krt19* were expressed in the epididymal tissue as well as in both cell lines, indicating that the cell lines represent epithelial origin and that ddR1 + R2 cells have gone through EMT only partially. Further, many of the keratins, such as basal cell-specific *Krt5*, *Krt14*, *Krt15* and *Krt17*, expressed in the Ctr epididymal tissue, were not expressed in the Ctr nor in the ddR1 + R2 cells, likely reflecting changes due to the immortalization of the original cell line. Interestingly, keratins *Krt4*, *Krt13*, *Krt20* and *Krt32*, which are not expressed in the epididymal tissue nor in the Ctr cells, were expressed in the ddR1 + R2 cells (Table 2). Partial shift towards EMT status in ddR1 + R2 organoids prompted us to analyse vimentin expression in Dicer1 cKO epididymides. A slight but significant upregulation (FC 1.3, p ≤ 0.01) of vimentin mRNA was also noted in Dicer1 cKO IS and Cap at the age of 40 days. Vimentin immunostaining from Dicer1 cKO epididymal sections showed occasional cells stained in the epithelium at the age of 40 days and even more pronounced epithelial staining at the age of 2 months (Fig. 4G and Fig. S6, respectively). All this indicates that while ddR1 + R2 cells and Dicer1 cKO epididymal epithelium still retain certain epithelial characteristics, their normal epithelial identity is severely compromised and cells gain EMT-related characteristics.

**Table 2:** Keratin RNA-seq values of Ctr and ddR1 + R2 cells (FKPM) and Ctr epididymal tissue (RPKM)

Keratin	Ctr (FKPM)	ddR1 + R2 (FKPM)	40 d Ctr IS (RPKM)	40 d Ctr Cap (RKPM)
*Krt4*	ND	1.4*	ND	ND
*Krt5*	ND	ND	50.5	73.5
*Krt7*	1.0	0.4*	33.0	45.3
*Krt8*	ND	2.5***	102.6	111.1
*Krt10*	2.1	3.2	3.3	2.4
*Krt13*	ND	1.7***	ND	ND
*Krt14*	ND	ND	26.0	32.9
*Krt15*	ND	ND	21.2	33.9
*Krt17*	ND	ND	13.5	1.9
*Krt18*	143.3	199.2	293.5	315.7
*Krt19*	2.2	1.6	65.9	224.1
*Krt20*	12.7	64.6***	ND	ND
*Krt23*	ND	1.2***	130.6	6.2
*Krt32*	ND	1.4***	ND	ND
*Krt80*	3.8	4.4	1.2	1.4
*Krt83*	2.3	3.2	ND	ND

*padj. ≤ 0.05

***padj. ≤ 0.001

### Lack of functional RUNX1 and RUNX2 disturbs *NOTCH* pathway activity

NOTCH signaling regulates differentiation and cellular lineage fate in various organs and has been linked to EMT e.g. in colorectal and hepatocellular cancers [45]. To study whether NOTCH pathway could play a role in EMT related changes observed in ddR1 + R2 organoids, we examined the RNA-seq data in this context. Interestingly, expression of several components of NOTCH signaling pathway was found altered in ddR1 + R2 organoids. The expression of *Notch2* (log2FC − 1.5, adj.p ≤ 5.1E−06), as well as of NOTCH downstream effectors *Hes1* (hes family bHLH transcription factor 1, log2FC − 1.0, adj.p ≤ 0.01) and *Hey1* (hairy/enhancer-of-split related with YRPW motif 1, log2FC − 3.5, adj.p ≤ 1.4E−16), was significantly down-regulated. Accordingly, while expressed only at a low level, *Notch3* mRNA was also significantly decreased in ddR1 + R2 organoids compared to Ctr (log2FC − 4.1, adj.p ≤ 1.9E−04). On the contrary, the expression of *Notch4* (log2FC 4.1, adj.p ≤ 4.8E−23) and *Hes6* (hairy and enhancer of split 6, log2FC 1.1, adj.p ≤ 6.7E−06) and NOTCH ligands *Jag2* (jagged 2, log2FC 1.3, adj.p ≤ 2.5E−08) and *Dll4* (delta like canonical Notch ligand 4, log2FC 0.8, adj.p ≤ 0.01) was increased.

Immunoblotting from pooled cultures of Ctr and ddR1 + R2 organoids showed that the expression level of NOTCH intracellular domain (NICD) of NOTCH1 was markedly reduced in ddR1 + R2 organoids compared to Ctr, and that the NICD of NOTCH2 was virtually absent in ddR1 + R2 organoids (Fig. 4H). Simultaneously, the protein level of HES1 was reduced (Fig. 4H). Together, these results suggest decreased NOTCH signaling in ddR1 + R2 organoid-like structures.

### Promoter analysis for RUNX binding sites

In order to explore whether some of the key regulators of the affected pathways could be direct transcriptional targets of RUNX, we utilized UCSC Genome Browser on the mouse genome (GRCm38/mm10) and visualized ReMap Atlas ChIP-seq data on RUNX1, 2 and 3 binding sites from all available mouse cell types and tissues (Fig. S7). The two MAPK pathway regulators with altered gene expression, *Spry1* and *Gpbar1*, both have RUNX binding sites in their promoter area and also in intron 2 of *Spry1* gene. For the EMT markers, both *Vim* and *Slit2* had multiple RUNX binding sites on the promoter and intron areas. Similarly, also *Notch1*, *Notch2, Notch3* and *Notch4* had RUNX binding sites in promoter and/or around the transcription start sites, suggesting that all of the above-mentioned factors could be direct RUNX target genes.

## Discussion

Transcription factors regulate tissue-specific cell differentiation and the maintenance of cellular identity. However, unlike for many other organs, apart from AR, the other essential TFs for the development and maintenance of the epididymis are not known. In this work, we have identified altogether 625 TFs expressed in the proximal mouse epididymis. Previously published mouse and human tissue transcription factor atlases reported on an average of 290 TFs detected in mouse tissues [8] and 1287 TFs in human [31] epididymis. In the mouse TF atlas, a smaller number of detected TFs is expected since proteins instead of mRNA were analyzed in that work. On the other hand, the two-fold higher number of TF mRNAs detected in the human epididymis compared to our work might reflect technical differences in sequencing and data analysis. In addition, we concentrated only on the most proximal regions of the mouse epididymis excluding corpus and cauda, whereas in the case of the human epididymis, the whole tissue was used. Separately analyzed IS and caput segments from 40-day-old samples allowed us to identify TFs exhibiting segment-specificity, and hence, likely to be of importance for proper segment identity. Among the few segment-specific TFs detected in this study, whose down-regulation in Dicer1 cKO epididymis coincided with the observed histological changes in epithelial epithelium, were the RUNX transcription factors. *RUNX1* expression has been shown to be regulated by AR in human prostate cancer and triple negative breast cancer cells in vitro [46, 47] and ReMap ChIP-seq data reveals AR binding sites on mouse *Runx1* and -*2* promoter and intronic areas. In addition, recent efferent duct ligation experiments suggest that testicular lumicrine factors regulate *Runx2* and to lesser extent *Runx1* in the mouse epididymis [48]. Indeed, it is possible that imbalanced AR signalling found in the Dicer1 cKO epididymis [17] contributes to impaired regulation of *Runx* expression in these tissues, but the link between the observations remains to be elucidated.

RUNX transcription factors regulate developmental processes, such as cell proliferation, differentiation, apoptosis and cell lineage specification. From the three mammalian RUNXs, RUNX1 is a key regulator of hematopoiesis [49], RUNX2 is a master regulator of skeletal development [50] whereas RUNX3 is important in immune cell development [51] and neurogenesis [52]. All three RUNX factors heterodimerize with core binding factor beta (*Cbfb*) and share the central DNA binding motif ‘PyGPyGGTPy’ [52]. Thus, it is not surprising that in certain cellular contexts, RUNX proteins may functionally compensate for each other [40, 53]. Both RUNX1 and -2 are expressed throughout the human epididymis with highest expression levels in cauda [54] and RUNX1 seems to function as a co-regulator of AR in the human epididymal epithelial cells from caput [55]. Our data from mouse epididymis demonstrate that RUNX1 and RUNX2 are expressed in the most proximal segment, the IS, and that both are required for the maintenance of proper epithelial identity of epididymal epithelial cells.

The differentiation of the most proximal epididymal segment, the IS, is initiated from developmental stage P15 onward when MAPK/ERK signaling pathway is activated by lumicrine factors [7]. The importance of MAPK/ERK signaling pathway for IS differentiation has been demonstrated using several knock-out or conditional mouse models, where the lack of ROS1 [56], PTEN [57], SRC [7] or MST1/2 [58] block ERK1/2 activation and result in reduced epithelial differentiation. In ddR1 + R2 cells, the MAPK/ERK signaling pathway appears hyperactivated with a significant increase in p-MEK levels. Accordingly, an increase was also seen in p-ERK levels, although it did not reach statistical significance. The regulators of ERK1/2 activation mentioned above were not significantly changed in ddR1 + R2 organoids. Instead, the down-regulation of *Spry1* and upregulation of *Gpbar1* likely account for the increased ERK activity. The observed up-regulation of multiple members within the Dusp gene family, particularly two ERK-specific DUSPs, namely *Dusp5* and *Dusp6*, serves as compelling evidence for hyperactive MAPK signaling upstream the action of DUSPs. Additionally, this up-regulation likely contributes to balancing the phosphorylation status of ERK. Hence, our data indicates that in the epididymal epithelium, RUNXs control the activity of MAPK signaling pathway via transcriptional regulation of *Spry1*, *Gpbar1* and several *Dusps*.

The best-known examples of MAPK/ERK-pathway overactivity come from various cancers in which the activity has been linked to cell proliferation, dedifferentiation, and a lack of apoptosis [59]. Furthermore, several studies have found that ERK activation in tumor cells leads to enhanced migration capability that can be alleviated by specific MAPK/ERK pathway inhibitors [60–62]. Thus, activated MAPK/ERK signaling could partly contribute to the enhanced mobility of ddR1 + R2 cells inside 3D Matrigel. In addition to examples from the context of cancers, renal epithelial MDCK-C7 cells were shown to dedifferentiate in response to constitutively active MAPK signalling [63]. Similar to our ddR1 + R2 cells, dedifferentiation of MDCK-C7 cells was accompanied by changes in cell morphology and increased vimentin expression [63]. However, whereas in kidney cells abolishment of cytokeratin expression was detected, ddR1 + R2 cells still express a variety of epithelial keratins, suggesting only partial or hybrid EMT of these epididymal epithelial cells. Moreover, MAPK/ERK pathway hyperactivity in mouse microglia cells has been previously reported to cause neurodegeneration [64]. Interestingly, in that study, GSEA hallmark analysis from RNA-seq data of affected microglia suggested EMT to be the most significantly affected process [64].

EMT is a part of the normal embryonal development in various tissues, but also frequently observed in epithelial carcinomas, typically associated with increased motility of tumor cells. RUNX family members have been linked to several cancers and have been shown to execute both tumor suppressor and oncogenic activities, strongly depending on the environment [65]. For example, in cervical cancer, RUNX1 overexpression was suggested to induce EMT and hence promote invasiveness of tumor cells [66]. However, in leukemia, the majority of the characterized *RUNX1* mutations, with the exception of AML-ETO fusion protein, diminish or abolish RUNX activity [67]. As a consequence, both an increase or a decrease of RUNX expression and functions may promote cancer progression. RUNX2 overexpression in breast and prostate cancer cells is associated with EMT and a specific metastatic phenotype known as osteomimicry, allowing cells to metastasize to bone [68]. For RUNX3, a tumor suppressor role has been reported in gastric cancer [69], contingently via a mechanism involving negative regulation of MMP9 [70] and vimentin [71]. In ddR1 + R2 cells, both the mRNA and protein levels of vimentin, a common mesenchymal marker, were up regulated. ReMap Atlas ChIP-seq data, encompassing a comprehensive analysis of RUNX binding sites across various mouse cell types and tissues, revealed the presence of RUNX binding sites on the *Vim* promoter. Interestingly, our data demonstrated Vimentin positive cells also in Dicer1 cKO epididymal epithelium and thus, it is likely that *Vim* is under direct negative transcriptional regulation by RUNX TFs in mouse epididymal cells.

In addition, opposing roles have recently been suggested for RUNX1 and RUNX2 in controlling the EMT of breast cancer stem cells [72]. In a tumor derived from human breast cancer stem cells grown in a mouse mammary fat pad, the expression of RUNX1 inhibits and the expression of RUNX2 and vimentin enhances the tumor growth. Interestingly, further studies with an inhibitor of CBFbeta-RUNX interaction resulted in EMT of breast cancer stem cells, suggesting that the loss of RUNX1 rather than increase of RUNX2 causes EMT in early stage breast cancer [72]. In a similar manner, RUNX1-CBFbeta interaction was shown to be a critical controller of lineage identity in normal mammary epithelial cells in vitro and its inhibition resulted in EMT [73]. In our studies, the simultaneous loss of RUNX1 and RUNX2 in the cells caused several significant changes which meet the criteria for EMT hallmarks [38]: cortical f-actin disorganization and up-regulation of vimentin expression (cytoskeletal remodeling), disorganized morphology of the organoids in 3D Matrigel (loss of apical-basal cell polarity and cell–cell adhesion weakening), delayed attachment to the plastic substratum (weakening of cell–matrix adhesion), altered growth pattern in 2D and 3D cultures (cell individualization and establishment of front-back polarity), and finally, enhanced motility through Matrigel matrix (acquisition of cell motility and basement membrane invasion). However, based on our data, it is impossible to determine whether one of the RUNXs could be solely responsible for the detected EMT characteristics. Yet, a deletion of either *Runx1* or *Runx2* alone did not result in such morphological changes that would support EMT.

SLIT2, identified from the MSigDB database’s EMT Hallmark gene set, has been implicated as a regulator of metastasis in lung cancer [74], colorectal cancer [44], and most recently in circulating tumor cells [75]. Significant down-regulation of SLIT2 expression in ddR1 + R2 cells prompted us to test whether the lack of secreted SLIT2 protein would be responsible for their increased motility. However, addition of recombinant SLIT2 protein into the culture medium did not affect cell mobility. Instead, a significant decrease was noted in the severity of the cellular protrusions reaching out from the ddR1 + R2 organoid body. In controls, organoids were generally very round with only occasional budding of tightly packed cells. In addition to its role in cell migration, the absence of SLIT2 has been proposed to diminish cell adhesion by increasing the expression of catenin beta 1 (*Ctnnb1*), and reducing the interaction between CTNNB1 and cadherin 1 (CDH1) [74]. Furthermore, the knock-down of SLIT2 in PC3 spheroids changed their morphology from highly to poorly organized [75]. Significantly increased *Ctnnb1* expression was also detected in ddR1 + R2 organoids. Therefore, it is likely that the augmented expression of *Ctnnb1*, impaired cell adhesion, and the disorganized 3D structure observed in ddR1 + R2 organoids could be partially attributed to the absence of SLIT2 in these cells. ReMap Atlas ChIP-seq data revealed RUNX binding sites on the *Slit2* promoter, suggesting that the observed lack of *Slit2* could be directly caused by the lack of RUNX action.

NOTCH signaling plays a fundamental role in many developmental processes, including EMT, by mediating cell–cell communication [45] and thus we wanted to see whether NOTCH signaling is affected in ddR1 + R2 cells. Indeed, we observed down-regulation of proteolytically activated NICD forms of NOTCH1 and NOTCH2 in these cells. Our RNA-seq data showed a consistent down-regulation of several components of the proteolytic system that is responsible for NOTCH activation, but these were mostly minor changes and not statistically significant. Nevertheless, a systematic reduction of the proteolytic components may have been able to affect availability of NOTCH1-NICD. However, NOTCH2-NICD was virtually absent in ddR1 + R2 cells. At the same time, the levels of *Notch2* and *Notch3* mRNAs were reduced showing that they are regulated by RUNX1 and RUNX2 either directly or indirectly also at the transcriptional level. Supported by the ReMap ChIP-seq data, it is indeed likely that RUNX molecules can directly bind to *Notch2* and *Notch3* promoters and positively regulate their transcription. The known examples of the crosstalk between RUNXs and NOTCH signaling pathway mostly show that NOTCH signaling regulates the expression of *Runx* or its transcriptional activity [76, 77]. However, in addition, RUNX1 has been shown to directly inhibit the expression of *NOTCH4* during human megakaryocytic differentiation [78]. This is in line with our data showing that *Notch4* was potently up-regulated in ddR1 + R2 cells lacking functional RUNX proteins. Moreover, ReMap ChIP-seq data demonstrated RUNX binding sites in the mouse *Notch4* promoter, suggesting a potential, direct transcriptional regulation by RUNX. Interestingly, NOTCH4 has been shown to inhibit proteolytic processing of full-length NOTCH1 [79], and thus, up-regulation of *Notch4* in ddR1 + R2 organoids may also contribute to the lower levels of NOTCH1-NICD. Finally, we showed that the expression of *Hes1* and *Hey1* was diminished and that of *Hes6* was increased in ddR1 + R2 cells. *Hes1* and *Hey1* are classical canonical target genes of NOTCH whereas *Hes6* was reported to be NOTCH-independent but targeting HES1 and thus inhibiting the effects of NOTCH signaling [80].

The role of NOTCH signaling in the epididymis is not well known. Constitutive over-expression of NOTCH1 NICD in the epididymis resulted in epithelial cell hyperplasia and a defect in epithelial cell differentiation [81]. However, in that model, hyperplasia led to the blockage of efferent ducts. Since testicular lumicrine regulation is necessary for epididymal function, the interpretation of the results is challenging. NOTCH signaling has pleiotropic effects affecting both cell proliferation and differentiation. Altogether, these results suggest that RUNX1 and RUNX2 have a promoting impact on the overall NOTCH signaling in epididymal cells and show that the lack of functional RUNX1 and -2 severely disturbs its function. Finally, while NOTCH signaling has been shown to promote EMT for example in colorectal cancer [82] and cardiac development [83], its putative opposite role in RUNX-mediated differentiation, maintenance of epithelial identity or shift to EMT in normal mouse epididymis, remains to be further clarified.

Taken together, we have identified transcription factor families expressed in the mouse epididymis and identified TFs whose altered expression pattern associated with observed loss of properly differentiated epithelial identity in Dicer1 cKO epididymides. Further, concentrating on the role of RUNX1 and -2, our in vitro analyzes demonstrates that both genes regulate several key signaling pathways in the epididymis and thus being essential for the maintenance and proper differentiation of the epididymal epithelium.

### Supplementary Information

Below is the link to the electronic supplementary material.

Supplementary file1 (DOCX 35 KB)

Supplementary file2 (XLSX 2051 KB)

Supplementary file3 (PDF 649 KB)

## Data Availability

RNA-seq data generated in this study were deposited in the GEO database under accession number GSE236105 and GSE236104. Microscopy data reported in this paper will be shared by the lead contact upon request. Any additional information required to reanalyze the data reported in this paper is available from the lead contact upon request.
