# Supplementary material for: RUNX transcription factors are essential in maintaining epididymal epithelial differentiation
Source: Cell Mol Life Sci. 2024 Apr 17;81(1):183. doi: 10.1007/s00018-024-05211-5 (PMC11023966; doi:10.1007/s00018-024-05211-5)
Supplement: Supplementary file 1 — Supplementary file1 (DOCX 35 KB) [file 18_2024_5211_MOESM1_ESM.docx]

**Table S1. The primary and secondary antibodies**

| **Antibody** | **Source** | **Identifier** |
| --- | --- | --- |
| AML1 (D4A6) Rabbit monoclonal | Cell Signaling Technology | Cat#8529 RRID:AB_10950225 |
| RUNX2 (D1H7) Rabbit monoclonal | Cell Signaling Technology | Cat#8486 RRID:AB_10949892 |
| Vimentin (D21H3) XP Rabbit monoclonal | Cell Signaling Technology | Cat#5741 RRID:AB_10695459 |
| Phospho-MEK1/2 (Ser217/221) (41G9) Rabbit monoclonal | Cell Signaling Technology | Cat#9154 RRID:AB_2138017 |
| MEK1 Rabbit polyclonal | Cell Signaling Technology | Cat#9124 RRID:AB_330804 |
| Phospho-p44/42 MAPK (Erk1/2) Rabbit monoclonal | Cell Signaling Technology | Cat#4377 RRID:AB_331775 |
| p44/42 MAPK (Erk1/2) Rabbit monoclonal | Cell Signaling Technology | Cat#4695 RRID:AB_390779 |
| Notch1 (D1E11) Rabbit monoclonal | Cell Signaling Technology | Cat#3608 RRID:AB_2153354 |
| Notch2 (D76A6) Rabbit monoclonal | Cell Signaling Technology | Cat#5732 RRID:AB_10693319 |
| HES1 (D6P2U) Rabbit monoclonal | Cell Signaling Technology | Cat#11988 RRID:AB_2728766 |
| Recombinant Anti-HES5 Rabbit monoclonal | Abcam | Cat#ab194111 |
| Anti-β-Actin clone AC-15 Mouse monoclonal | Sigma-Aldrich | Cat#A1978 RRID:AB_476692 |
| ZO-1 Rat monoclonal | Ref Breton et al |  |
| ZO-2 Rabbit polyclonal | Cell Signaling Technology | Cat#2847 RRID:AB_2203575 |
| ZO-3 (D57G7) XP^®^ Rabbit monoclonal | Cell Signaling Technology | Cat#3704 RRID:AB_2203606 |
| Claudin 1 Rabbit polyclonal | Invitrogen | Cat#51-9000 RRID:AB_2533916 |
| Claudin 3 Rabbit polyclonal | Invitrogen | Cat#34-1700 RRID:AB_2533158 |
| Claudin 4 (ZMD.306) Rabbit polyclonal | Invitrogen | Cat#36-4800 RRID:AB_2533262 |
| Anti-mouse IgG, HRP-linked Horse polyclonal | Cell Signaling Technology | Cat#7076 RRID:AB_330924 |
| Anti-rabbit IgG, HRP-linked Goat polyclonal | Cell Signaling Technology | Cat#7074  RRID:AB_2099233 |
| Anti-Rabbit IgG (H+L) Alexa Fluor™ 594, Goat polyclonal | Invitrogen | Cat#A-11037 RRID:AB_2534095 |
