# Supplementary material for: RUNX transcription factors are essential in maintaining epididymal epithelial differentiation
Source: Cell Mol Life Sci. 2024 Apr 17;81(1):183. doi: 10.1007/s00018-024-05211-5 (PMC11023966; doi:10.1007/s00018-024-05211-5)
Supplement: Supplementary file 3 — Supplementary file3 (PDF 649 KB) [file 18_2024_5211_MOESM3_ESM.pdf]

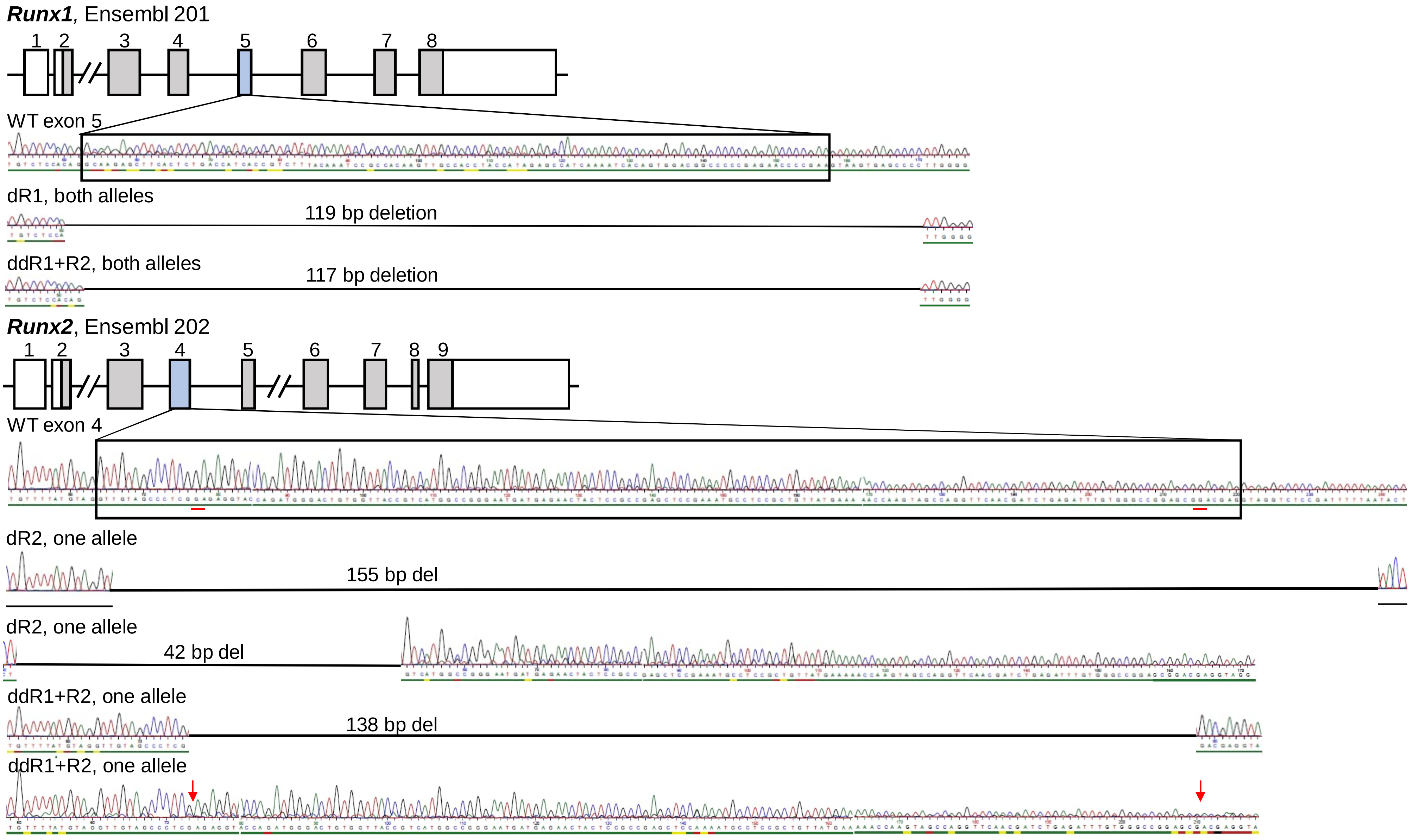

Runx1, Ensembl 201
1
2
3
4
5
6
7
8
WT exon 5
dR1, both alleles
119 bp deletion
ddR1+R2, both alleles
117 bp deletion
Runx2, Ensembl 202
1
2
3
4
5
6
7
8
9
WT exon 4
dR2, one allele
155 bp del
dR2, one allele
42 bp del
ddR1+R2, one allele
138 bp del
ddR1+R2, one allele

Figure S1. Characterization of *Runx1* and *Runx2* mutations in mE-Cap18 cells. Schematic picture of the gene structure of *Runx1* and *Runx2* with untranslated regions (UTR) areas white, coding sequence gray and exon coding DNA binding domain blue. Sequence from exon 5 (*Runx1*) and exon 4 (*Runx2*) is marked with box in WT sequence. Different deletions in sequences are marked for cell lines with only *Runx1* (dR1) or *Runx2* (dR2) deletion and with both deletions (ddR1+R2). For *Runx2*, point mutations, deletions of nucleotide G in both sites, in one allele of ddR1+R2 cell line are marked with arrows and corresponding nucleotides are underlined in WT sequence.

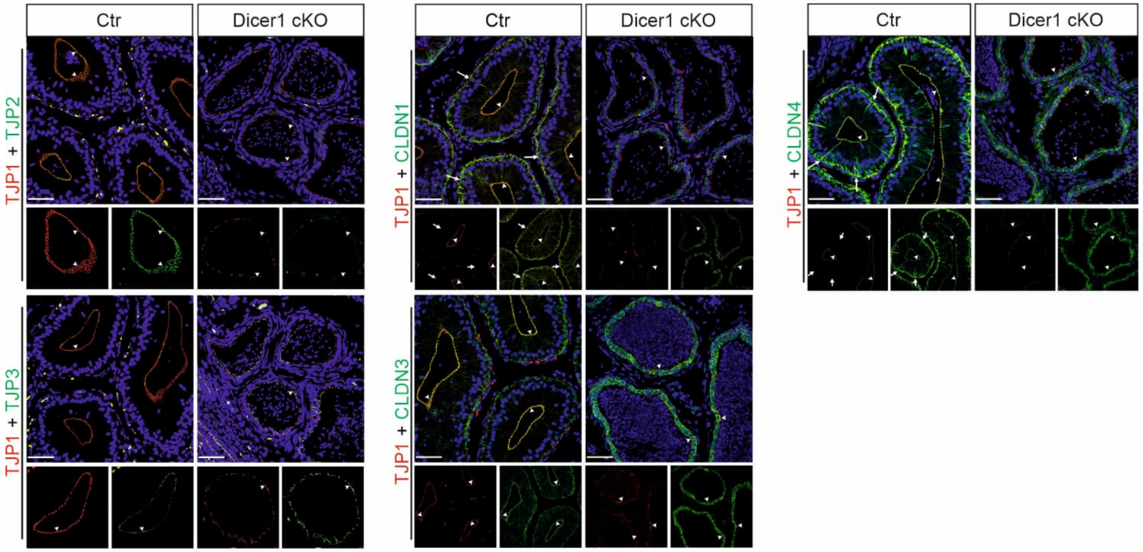

Figure S2. Immunofluorescent staining of tight junction proteins in control (Ctr) and Dicer1 cKO epididymides. Tight junction proteins (TJPs); TJP1 (red), TJP2 (green) and TJP3 (green), and claudins (CLDN, green); CLDN1, CLDN3 and CLD4 were stained in adult 2-month-old Ctr and Dicer1 cKO epididymides. In Ctr TJP1, TJP2 and TJP3 were highly expressed and largely colocalized in the tight junctions in the apical side of the epithelium, whereas in Dicer1 cKO epididymides the expression of TJP1 and TJP2 was markedly reduced, TJP2 being almost undetectable in the tissue sections (left panel, arrowheads). TJP1 staining was discontinuous, suggesting problems in the apical tight junctions of Dicer1 cKO mice. Contrary to TJP1 and -2, TJP3 seemed to be upregulated in Dicer1 cKO, but with similarly discontinuous staining as TJP1 (left panel, arrowheads). CLDN1, -3 and -4 were strongly colocalized with TJP1 in apical tight junctions (middle and right panels, arrowheads) and were detected in the basolateral membrane of epithelial cells in Ctr. CLDN1 and -4 were also detected throughout the plasma membrane of basal cells (arrows). In contrast, no colocalization with TJP1 was observed for any of the claudins in Dicer1 cKO epididymides apical junctions (middle and right panels, arrowheads). Instead the expression of claudins was detected throughout the cytoplasm and basolateral membranes of the Dicer1 cKO epithelium. DNA; blue. Yellow color indicates colocalization. Arrow; basal cell. Scale bars 50 µm.

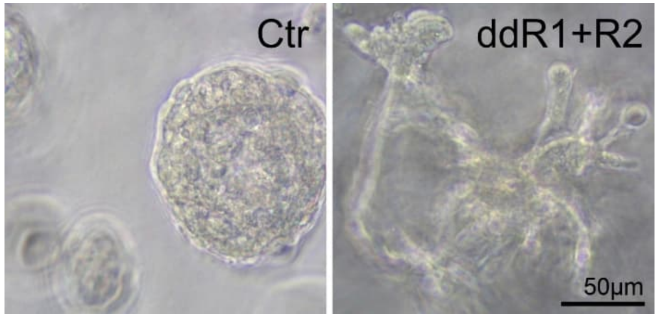

Figure S3. Representative phase-contrast images of the 3D organoid-like structures used in the RNAseq analysis. RNA was harvested on day 12 of culture in Matrigel. Scale bar 50 µm.

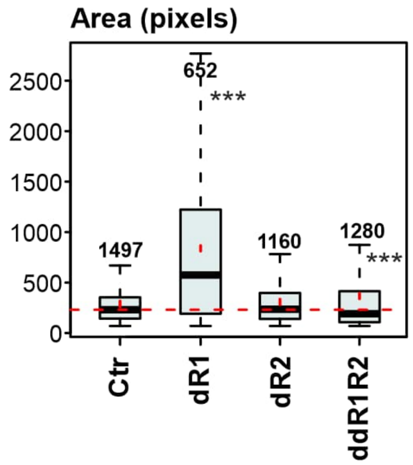

Figure S4. Morphometric analysis of organotypic cultures (*Area* measurement). AMIDA analysis from confocal microscope images of organoid-like structures at day 10 of culture. *Area* describes the size of an individual object. Data visualization and statistical analysis were performed in the R-software environment (www.r-project.org) using Bonferroni-corrected *t*-tests against Ctr. The number above the box is the number of individual objects analyzed across replicate wells. Data presented as a box and whisker plot (median, black line; mean, red spot). *** $p \leq 0.001$.

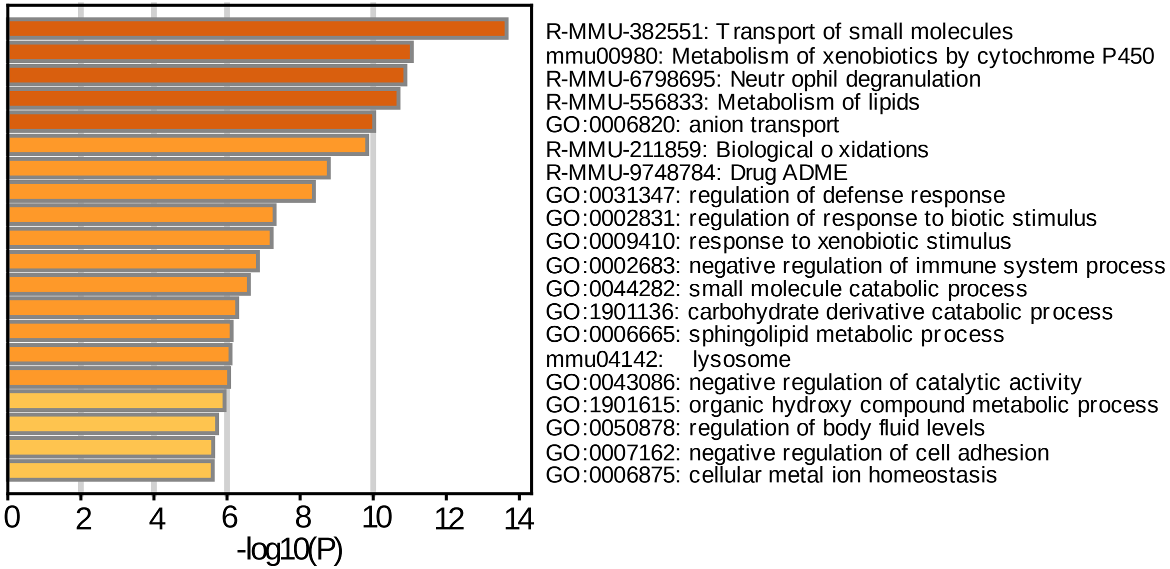

Figure S5. Lack of RUNX1 and RUNX2 affects several signaling pathways in organotypic cultures. Top twenty GO terms associated with up-regulated DE genes in ddR1+R2 organoid-like structures.

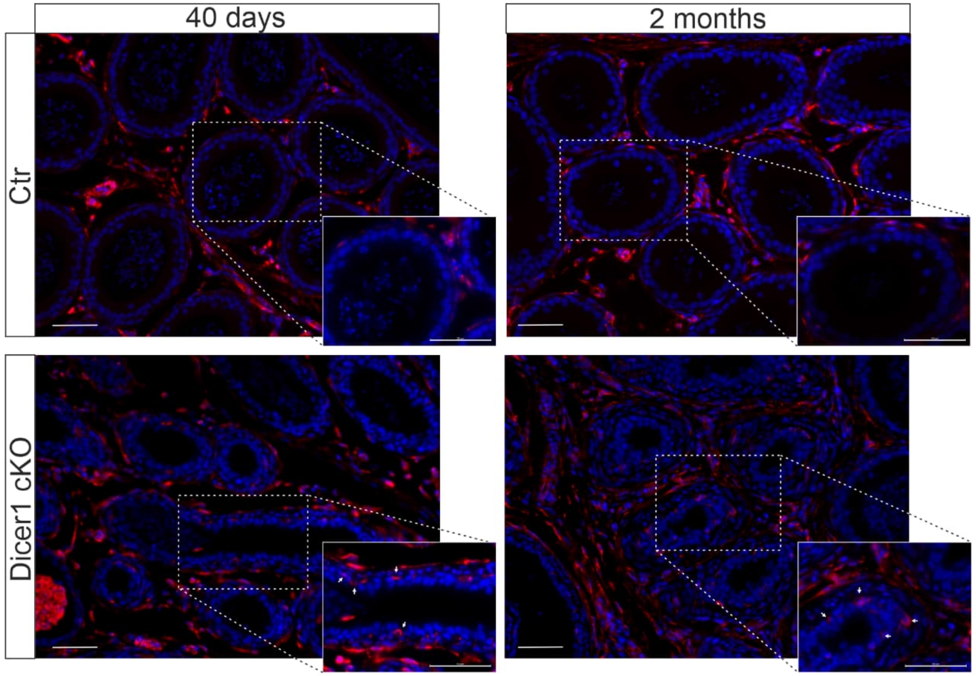

Figure S6. Immunofluorescent staining of vimentin in control (Ctr) and Dicer1 cKO epididymides. Vimentin (red) was stained in 40-day-old and adult 2-month-old Ctr and Dicer1 cKO epididymides. In Ctr VIM was highly expressed in the mesenchymal cells between the tubulus cross sections, whereas in Dicer1 cKO epididymides also some epithelial cells express VIM (inserts, arrows). DNA; blue. Scale bars 50 µm.

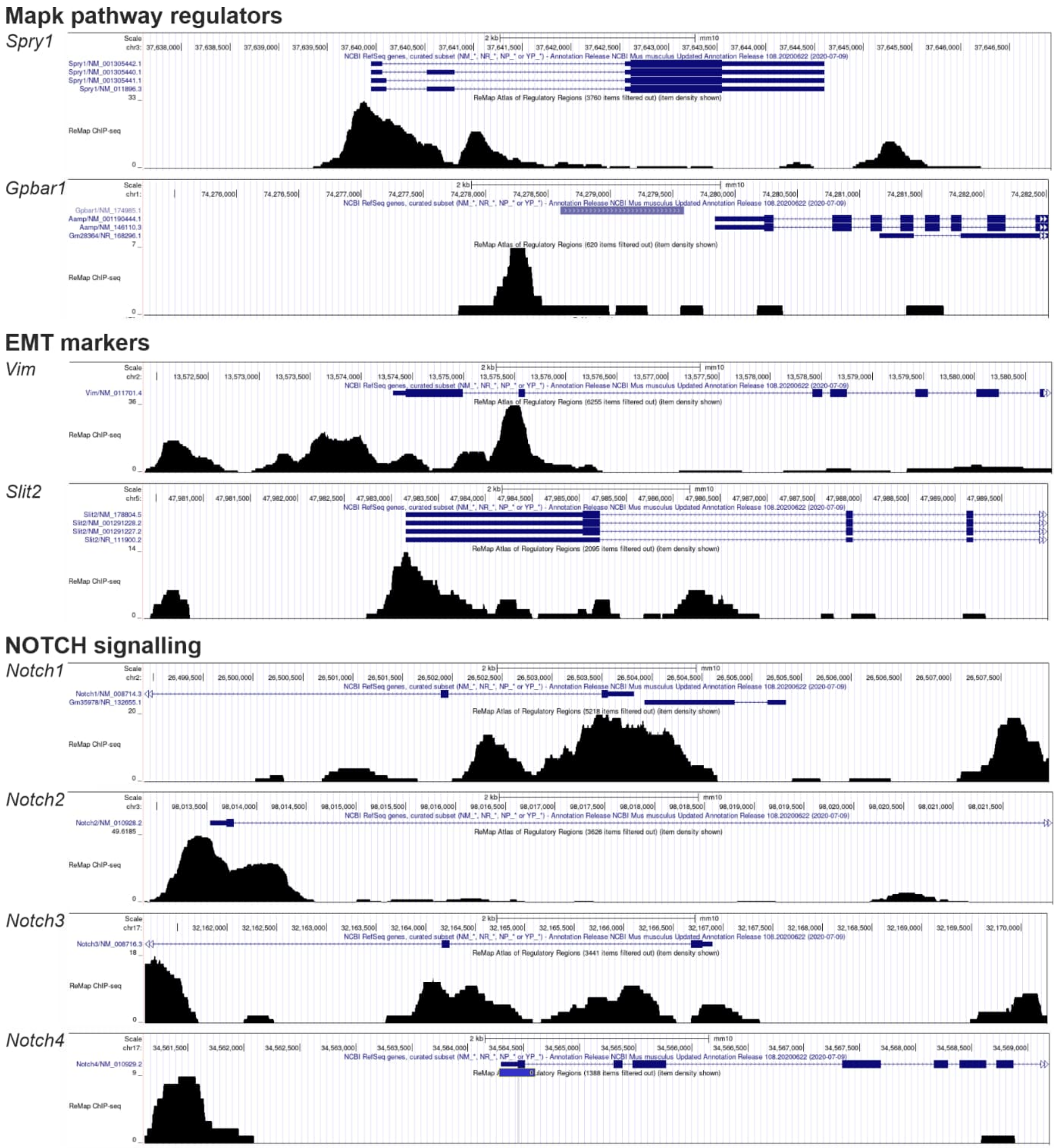

Figure S7. ReMap Atlas ChIP-seq data on RUNX1, -2 and -3 binding sites on mouse tissues.
Detected RUNX binding sites for MAPK pathway regulators sprouty RTK signaling antagonist 1 (Spry1), G protein-coupled bile acid receptor 1 (Gpbar1); markers for epithelial to mesenchymal transition vimentin (Vim) and slit guidance ligand 2 (Slit2) as well as NOTCH signaling receptors Notch1, Notch2, Notch3 and Notch4 visualized using UCSC Genome Browser on mouse genome (GRCm38/mm10).
